# Identification of MicroRNAs as Potential Prognostic Markers in Ependymoma

**DOI:** 10.1371/journal.pone.0025114

**Published:** 2011-10-28

**Authors:** Fabricio F. Costa, Jared M. Bischof, Elio F. Vanin, Rishi R. Lulla, Min Wang, Simone T. Sredni, Veena Rajaram, Maria de Fátima Bonaldo, Deli Wang, Stewart Goldman, Tadanori Tomita, Marcelo B. Soares

**Affiliations:** 1 Cancer Biology and Epigenomics Program, Children's Memorial Research Center and Department of Pediatrics, Northwestern University's Feinberg School of Medicine, Chicago, Illinois, United States of America; 2 Department of Pathology and Laboratory of Medicine, Children's Memorial Hospital, Chicago, Illinois, United States of America; 3 Pediatric Neurosurgery, Children's Memorial Hospital, Chicago, Illinois, United States of America; 4 Pediatric Neuro-oncology, Children's Memorial Hospital, Chicago, Illinois, United States of America; 5 Biostatistics Research Core, Children's Memorial Hospital, Chicago, Illinois, United States of America; Biomedical Research Institute, United States of America

## Abstract

**Introduction:**

We have examined expression of microRNAs (miRNAs) in ependymomas to identify molecular markers of value for clinical management. miRNAs are non-coding RNAs that can block mRNA translation and affect mRNA stability. Changes in the expression of miRNAs have been correlated with many human cancers.

**Materials and Methods:**

We have utilized TaqMan Low Density Arrays to evaluate the expression of 365 miRNAs in ependymomas and normal brain tissue. We first demonstrated the similarity of expression profiles of paired frozen tissue (FT) and paraffin-embedded specimens (FFPE). We compared the miRNA expression profiles of 34 FFPE ependymoma samples with 8 microdissected normal brain tissue specimens enriched for ependymal cells. miRNA expression profiles were then correlated with tumor location, histology and other clinicopathological features.

**Results:**

We have identified miRNAs that are over-expressed in ependymomas, such as miR-135a and miR-17-5p, and down-regulated, such as miR-383 and miR-485-5p. We have also uncovered associations between expression of specific miRNAs which portend a worse prognosis. For example, we have identified a cluster of miRNAs on human chromosome 14q32 that is associated with time to relapse. We also found that miR-203 is an independent marker for relapse compared to the parameters that are currently used. Additionally, we have identified three miRNAs (let-7d, miR-596 and miR-367) that strongly correlate to overall survival.

**Conclusion:**

We have identified miRNAs that are differentially expressed in ependymomas compared with normal ependymal tissue. We have also uncovered significant associations of miRNAs with clinical behavior. This is the first report of clinically relevant miRNAs in ependymomas.

## Introduction

Ependymoma is a common pediatric central nervous system (CNS) tumor that is believed to originate from ependymal cells located in the lining of ventricular surfaces in the brain [1]. Outcomes for children with ependymoma have not significantly changed over the past several decades despite advances in neurosurgical techniques and adjuvant therapy. Attempts to predict patient outcome have been limited by the heterogeneous clinical behavior of patients with ependymoma and many contradicting studies of existing clinical, pathologic and biologic prognostic markers. Gross total resection remains the single most important predictor of overall and relapse-free survivals in pediatric ependymoma [2]. Treatments currently used in the clinics, such as radiotherapy and standard-dose chemotherapy, have resulted in improved outcome, but we are in need of biomarkers for better diagnosis, prognosis and for management of disease progression. Histopathologic analysis is not predictive of clinical behavior, such as the likelihood of recurrence [1], [2]. Hence, there is a need for studies aimed at the identification of molecular markers of clinical value.

MicroRNAs (miRNAs) are short non-coding RNAs (approximatelly 20 nucleotides) that act by blocking messenger RNA translation, having a major impact in the regulation of protein-coding gene networks and pathways [3]. They also work as master regulators of gene expression, and they have been associated with a wide range of biological processes, including differentiation, proliferation and apoptosis [4]. miRNAs have also been implicated in a variety of diseases, including different types of cancers. In general, miRNAs that are over-expressed in tumors contribute to oncogenesis by down-regulating tumor supressor genes (e.g. miR-21 regulating PTEN) [5], whereas those with reduced expression in tumors may cause derepression or increased expression of an oncogene (e.g. let-7 regulating RAS, MYC and HMGA2) [6], [7]. Cancer-associated miRNAs have also been identified as regulators of the p53 gene network [8], and have been used as classifiers to subgroup different tumor types that are not easily categorized by traditional pathology and histology [9]. Expression profile analyses have revealed miRNAs that can either promote [10] or strongly suppress tumor metastasis [11]. Therefore, there is strong evidence that miRNAs are implicated in different steps of tumorigenesis, from tumor initiation, tumor maintenance, and progression to metastasis. However, limited data on miRNA expression in ependymoma has been previously published.

In this study, we have compared the miRNA expression profile of thirty-four ependymoma samples to normal brain controls by TaqMan Low Density arrays (TLDAs). Normal brain tissue further enriched with ependymal cells were used as normal controls. First, we analyzed and compared the correlation of miRNA expression profiles using paired frozen (FT) and paraffin (FFPE) tissue from the same patients, which was done as a “proof of principle” to demonstrate that paraffin-embedded specimens could be used reliably for this type of analysis. Once verified, we proceeded to analyze the miRNA expression profiles of multiple ependymomas using FFPE blocks. We correlated miRNA expression profiles with clinicopathologic parameters, morphological features and location of the tumor in the brain. Using this strategy, we have been able to generate a miRNA signature that can distinguish normal ependymal cells from ependymomas. Interestingly, we have identified a miRNA, miR-485-5p, that is down-regulated in ependymomas and putatively targets members of the TGFbeta family of genes. Using both univariate and multivariate Cox regression analysis, we were able to identify miRNAs whose differential expression in ependymoma was associated both with time to relapse and overall survival. More specifically, we have identified a cluster of miRs on human chromosome 14q32 that correlates to Time to Relapse by both Cox regression analysis and Kaplan-Meier curves. One such miR, miR-203, was identified as an independent marker for tumor relapse even when factoring in the clinicopathologic variables that are currently utilized in the clinics for prediction of clinical behavior. In addition, three miRNAs (let7d, miR-596 and miR-367) were identified as better predictors for Overall Survival.

## Materials and Methods

### Patients and samples

Tumor samples and normal brain specimens were collected retrospectively from the Falk Brain Tumor Tissue bank at Children's Memorial Hospital (Chicago, IL) under an IRB-approved protocol. Both FFPE and frozen specimens of ependymoma were obtained from pediatric patients treated between 1997 and 2010. Initially, we collected 45 tumor FFPE blocks and profiled microRNAs for all of them. From these, just 34 samples passed our pipeline for analysis based on RNA quality, quantity and also after a stringent analysis using the internal controls from the TLDA microRNA arrays. Normal specimens in our study represent core biopsies of the lateral ventricular lining of the basal ganglia and thalamus of patients who died from non brain-related illnesses, which were microdissected and enriched for ependymal cells. Clinicopathologic information such as age, tumor location, time to relapse and overall survival for each ependymoma specimen were also collected by retrospective medical record review (Table 1).

**Table 1:** Clinicopathologic characteristics of the ependymoma samples used in this study.

Sample	Sex	Age atDiagnosis(years)	Tumor Location	Specimen	TreatmentPrior to Surgery	Age at Surgery (years)	WHO Grade*	SpecimenTime toRelapse(years)	Patient Status	Follow-up(years)*
1A	F	8.2	ST	Recurrent	RT	17.2	II	0.9	DOD	10.6
1B	F	8.2	ST	Recurrent	C/RT	18.1	II	0.2	DOD	10.6
2	M	7.0	IT	Recurrent	RT	16.2	II	0.6	DOD	10.0
3A	F	16.4	ST	Primary	None	16.4	III	0.4	NED	8.8+
3B	F	16.4	ST	Recurrent	None	16.8	III	1.5	NED	8.8+
3C	F	16.4	ST	Recurrent	RT	18.3	II	NR	NED	8.8+
4A	M	1.2	IT	Recurrent	C	6.2	III	1.2	DOD	11.2
4B	M	1.2	IT	Recurrent	C	7.5	II	1.7	DOD	11.2
5	M	2.2	ST	Recurrent	None	3.9	II	NR	NED	13.4+
6A	F	9.2	IT	Recurrent	C/RT	13.2	II	0.5	DOD	6.8
6B	F	9.2	IT	Recurrent	C/RT	13.8	II	1.1	DOD	6.8
6C	F	9.2	IT	Recurrent	C/RT	14.9	II	0.8	DOD	6.8
7	F	7.6	IT	Primary	None	7.6	II	1.2	DOD	2.4
8	F	9.7	IT	Recurrent	C/RT	14.6	II	NR	NED	13.1+
9	M	5.2	IT	Recurrent	C/RT	6.6	II	2.2	NED	7.1+
10	F	0.2	IT	Primary	None	0.2	III	0.6	DOD	0.6
11	M	0.9	IT	Primary	None	0.9	II	1.0	DOD	7.1
12A	F	1.1	IT	Primary	None	1.1	III	1.8	DOD	4.0
12B	F	1.1	IT	Recurrent	C/RT	3.0	III	0.2	DOD	4.0
12C	F	1.1	IT	Recurrent	C/RT	4.5	III	0.3	DOD	4.0
13	F	6.3	ST	Recurrent	None	8.1	III	1.2	NED	10.7+
14	F	15.4	IT	Primary	None	15.4	II	0.3	DOD	2.5
15	F	1.9	IT	Primary	None	1.9	III	0.4	DOD	0.5
16	M	4.1	IT	Recurrent	C/RT	11.0	III	0.2	AWD	10.3+
17	M	1.3	IT	Primary	None	1.3	II	1.2	DOD	1.4
18	F	11.1	IT	Primary	None	11.1	II	NR	NED	8.4+
19	F	1.4	IT	Primary	None	1.5	II	NR	NED	8.4+
20	M	5.8	IT	Primary	None	5.8	II	2.5	DOD	3.4
21	M	12.8	IT	Recurrent	RT	14.5	II	0.8	DOD	3.5
22	M	0.8	IT	Primary	None	0.8	II	1.0	DOD	6.8
23	M	0.8	IT	Recurrent	C/RT	5.8	II	0.9	DOD	6.8
24	M	2.5	IT	Recurrent	C/RT	11.4	II	1.0	DOD	11.0
25	F	11.1	ST	Primary	None	11.1	III	NR	NED	6.4+
26	F	5.2	IT	Primary	None	5.2	III	NR	NED	4.9+

**Abbreviations:** IT, Infratentorial; ST, Supratentorial; C, Chemotherapy treatment; RT, Radiotherapy treatment; NR, No Relapse to date; AWD, Alive With Disease; NED, No Evidence of Disease; DOD, Dead as a result Of the Disease;

*Based on standard WHO (World Health Organization) classification for ependymomas.

**Note**: The letters represent different samples from the same patient.

### RNA extraction

For frozen tissue, a sample of approximately 0.5 cm^3^ in dimension was used for RNA extraction. Total RNA was extracted using a Trizol standard protocol (Invitrogen, Carlsbad, CA). For FFPE samples, total RNA was isolated from five to twenty 10 µm-thick tissue sections using the Ambion RecoverAll kit (Ambion/Applied Biosystems, Foster City, CA) according to the manufacturer's instructions. Total RNA quantity and quality were evaluated using a spectrophotometer (Nanodrop ND-1000, Thermo Scientific, Wilmington, USA) and agarose gel electrophoresis.

### Quantification and confirmation of microRNA expression

TaqMan® Low Density Arrays (TLDAs) v1.0 (Applied Biosystems, Foster City, CA) were used to quantify the expression levels of 365 mature miRNAs and controls. These microRNAs were selected based on the customized array manufactured by Applied Biosystems (version 1.0). It is important to note that this TLDA array contained some microRNAs already associated to human cancers. A complete list of the microRNAs that are present in the array is shown on Table S1. Reverse Transcriptase (RT) reactions were done according to the manufacturer's instructions in a 7900HT Fast Real-Time PCR system as described by Raymond *et al.*, 2005 and Chen *et al*., 2005 [12], [13]. RNA was purified as described above and each RT reaction contained purified total RNA and reagents from the TaqMan® microRNA Reverse Transcription Kit. Reactions were run in a 384-well TLDA block at 94.5°C for 10 min, followed by 40 cycles at 97°C for 30 s and 60°C for 1 min. Individual miRNA expression analysis for miR-135a, miR-17-5p, miR-485-5p, miR-383 and miR-34a were performed in triplicate in 96 well plates using the TaqMan® Individual miRNA assays (Applied Biosystems) according to the manufacture's instructions.

### Bioinformatic analysis and microRNA target identification

Putative targets for all differentially expressed miRNAs listed on Table S2 were predicted using combined bioinformatics methods and literature search. Both the GOmir software (http://bioacademy.gr/bioinformatics/projects/GOmir), and the GeneGo suite (http://www.genego.com/) were utilized. The candidates that were selected by these analyses were then ascertained using microarray data from gene expression (Illumina, CA, USA). The values of expression were normalized and ependymoma samples were averaged against all normal samples.

### Statistical analysis

For all miRNA quantification experiments, cycle threshold (Ct) values greater than 35 were excluded. Values were then normalized against the expression levels of RNU48 and Delta Ct values were calculated using Real-Time StatMiner software (Integromics TM, Philadelphia, PA). Multi-Experiment Viewer v4.1 (http://www.tm4.org/mev.html) was used to generate unsupervised and supervised hierarchical clusterings based on support tree average linkage with Pearson and Eucledian correlations. The Empirical Bayes Moderated t-test was used by the StatMiner software (Integromics, TM) to generate Delta-Delta Ct values for each comparison. P-values were adjusted using the FDR *Benjamini-Hochberg* method, and significance was attributed if FDR<0.05 for all analyses. Fold changes were calculated by StatMiner using the formula Log_10_RQ = Log_10_ 2^−Δ(ΔCT)^ (Log_10_RQ = 0 corresponds to no expression change; Log_10_RQ = 1 means that the test sample is expressed at a level 10 times greater than that of the calibrator sample; Log_10_RQ = −1 means that the test sample is expressed at a level 10 times lower than that of the calibrator sample). MiRNA expression levels of normal controls and tumor tissues were also evaluated using the analysis of variance (ANOVA).

miRNA expression analyses of association with clinical variables were conducted using Wilcoxon Rank Sum test. Time to relapse or recurrence-free survival, and overall survival (OS) were analyzed for different groups of clinical variables with the log-rank test and Kaplan-Meier curves were thus generated. A multivariate Cox regression model was used to select significant clinical variables and miRNAs to predict time to relapse and overall survival time in the patient cohort herein investigated. A survival tree analysis method was used to determine thresholds of cut-off points for selected miRNA expression in order to generate Kaplan-Meier curves for time to relapse. All analyses were conducted using the SAS 9.2 software (SAS Institute Inc., Cary, NC, USA), and the R package (R Development Core Team, Vienna, Austria, http://www.R-project.org).

## Results

### Characteristics of the patients and samples

Two frozen tumors and thirty-four FFPE specimens from twenty-six patients were collected and analyzed. The clinicopathologic characterstics of the samples used in this study are presented in Table 1. Of the thirty-four specimens, eight samples represented recurrent tumors from five patients. Twenty-six samples were from the infratentorial location (IT) and eight samples were supratentorial (ST). Fourteen samples were primary tumors and twenty represented recurrent tumors. The median age of our patient cohort was 7.1 years (range 0.2 to 19.2 years). At the time of data censoring, 9 patients were still alive and the remainder had succumbed to the disease.

We sought to investigate the correlations of the clinicopathologic variables of the patients used in our study. First, we evaluated the differences in patient age at diagnosis (<3 or >3 year of age), location (infratentorial versus supratentorial), prior treatment, extent of resection, WHO grade, patient outcome (succumbed to the disease, or alive with or without the disease) and tumor type (primary versus recurrent) by Kaplan-Meier Curves. We have found statistically significant differences in overall survival for resection (p = 0.0004), tumor type (p = 0.01) and tumor location (p = 0.01) (Figure S2). On the other hand, we were not able to identify any variable exhibiting significant correlation with time to relapse. However, a trend in variables such as resection and prior treatment (p = 0.07) was observed (Figure S3). For all the other variables both overall survival and time to relapse were not statistically significant (data not shown).

### microRNA expression profiling of ependymoma samples

In order to evaluate the correlation between frozen and paraffin tissue from the same patient, we profiled the expression levels of 365 miRNAs in paired samples of two patients. The first pair of specimens had been archived for 5 years and showed a high correlation between FFPE and frozen tumor (r = 0.99, p<0.05); the second pair (archived for 10 years) also exhibited a strong correlation (r = 0.94, p<0.05, data not shown).

**Figure 1:**
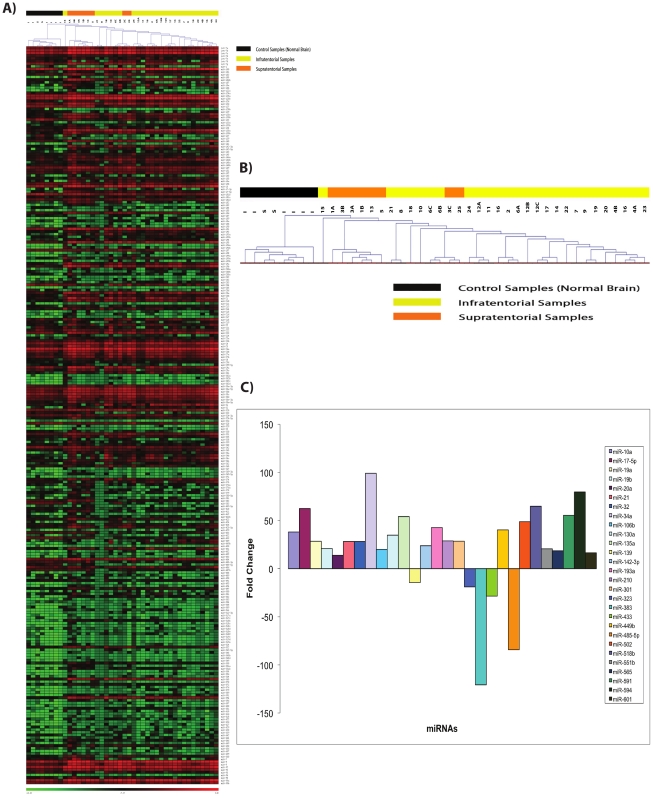
Heat Map representations for miRNA expression in ependymomas and normal controls. (**A**) Unsupervised Hierarchical Clustering of all normal brain control samples against the ependymomas used in this study. The clustering and tree are based on Pearson's correlation and were generated by the MeV 4.1 software according to Delta Ct values. The miRNAs that are not represented in this Figure were not detected in any of the groups. (**B**) Higher magnification of the Unsupervised Hierarchical Clustering from panel A with the supporting tree showing the separation between normal samples and ependymomas from different locations. Black, Normal controls; Yellow, Infratentorial samples; Orange, Supratentorial samples. NB-I, Normal ependymal cells from infratentorial location; NB-S, Normal ependymal cells from Supratentorial location. (**C**) Histogram graphical representation of the statistically differentially expressed miRNAs when comparing all normal samples against the ependymoma samples used in this study. The values in the Y axis are of fold changes based on the StatMiner software. Twenty-eight miRNAs are differentially expressed - twenty-three miRNAs are over-expressed and five miRNAs are downregulated (p≤0.01; FDR<0.05).

We then proceeded to evaluate miRNA expression in paraffin samples from thirty-four ependymomas and compared them to eight samples derived from normal brain tissue enriched for ependymal cells (6 infratentorial and 2 supratentorial). Unsupervised hierarchical clustering of miRNA expression levels revealed that normal brain and ependymoma samples cluster independently (Figures 1A and 1B). On the other hand, many of the supratentorial ependymomas cluster together, separated from the infratentorial tumors (Figure 1B). However, two of the supratentorial tumors were disparate from the main supratentorial branch (Figure 1B).

Twenty-eight miRNAs were identified as differentially expressed in ependymomas compared to normal controls (Figure 1C, Table 2, p≤0.01, FDR<0.05). We have verified that miR-34a and miR-135a are over-expressed in our samples when compared to normal controls. MiR-485-5p was identified in these analyses as down-regulated in ependymomas when compared to normal controls (Table 2 and Figure 1C). Using only differentially expressed miRNAs, we generated a supervised hierarchical clustering, which confirms that normal samples and tumors cluster independently (Figure S1A). Individual assays were then performed to confirm the over-expression (e.g. miR-135a and miR-17-5p), and the down-regulation (e.g. miR-383 and miR-485-5p) of a few selected miRNAs (Figure S1B).

**Table 2:** miRNAs differentially expressed in ependymomas (p≤0.01, FDR<0.05).

miRNA	E. vs C.	I. vs IC.	S. vs SC.	I. vs S.
**miR-7**	-	-	-	−46.0
**miR-10a**	38.1	-	-	-
**miR-15a**	-	53.2	-	-
**miR-17-5p** §	62.4	109.6	-	-
**miR-19a**	28.4	33.1	-	-
**miR-19b**	20.9	25.6	-	-
**miR-193a**	-	58.0	-	-
**miR-20a**	13.9	18.5	-	-
**miR-21**	28.2	31.8	-	-
**miR-23a**	-	14.5	-	-
**miR-30b**	-	12.9	-	-
**miR-31**	-	42.1	-	73.8
**miR-32**	28.2	57.2	-	-
**miR-34a** §	99.0	80.2	198.8	-
**miR-106b**	20.0	17.9	-	-
**miR-107**	-	-	-	−18.6
**miR-124a**	-	-	-	−61.1
**miR-128b**	-	-	−47.6	-
**miR-130a**	34.8	43.9	-	-
**miR-130b**	-	14.3	-	-
**miR-135a** §	54.1	70.5	-	-
**miR-139**	−14.5	-	-	-
**miR-142-3p**	23.8	23.5	-	-
**miR-155**	-	14.1	-	-
**miR-183**	-	-	-	−29.0
**miR-193a**	42.7	58.0	-	-
**miR-210**	28.8	32.1	-	-
**miR-301**	28.5	35.5	-	-
**miR-323**	−18.9	−23.1	-	-
**miR-339**	-	-	-	−11.5
**miR-376a**	-	-	-	−15.8
**miR-383** §	−120.7	−156.2	-	-
**miR-433**	−28.5	−55.3	-	-
**miR-449b**	40.3	54.1	-	-
**miR-485-5p** §	−84.2	−197.8	-	-
**miR-502**	48.8	62.9	-	-
**miR-518b**	64.8	44.0	218.3	-
**miR-551a**	-	-	194.4	−25.1
**miR-551b**	20.6	27.2	-	-
**miR-565**	18.6	17.6	-	-
**miR-591**	55.4	32.3	305.1	-
**miR-594**	79.6	79.2	83.7	-
**miR-601**	16.5	15.2	-	-

**Abbreviations:** E, Ependymomas; C, All Normal Controls; I, Infratentorial Tumors; IC, Infratentorial Normal Controls; S, Supratentorial Tumors; SC, Supratentorial Normal Controls.

§ miRs that were confirmed by individual assays.

**Note:** The cut off used in these analyses was 10 fold up and/or down with a p≤0.01 according to individual confirmation assays.

### Bioinformatic target predictions of differentially expressed microRNAs

We have utilized both GoMir and GeneGo for target identification of specific miRNAs that were significantly differentially expressed in the comparisons shown in Table 2. Based on gene expression data we have also evaluated the miRNA targets that were predicted from the GeneGo networks (Table S2). Using this strategy, we have identified putative targets for miRNAs that were found to be down-regulated and over-expressed in ependymomas. Accordingly, it is noteworthy that - as an example - components of the TGFbeta pathway were identified as over-expressed in ependymomas, and they are putative targets for miR-485-5p (down-regulated in our analysis) (Table 2, Figure 1C and Figure S1B).

### microRNAs as potential biological markers in ependymomas

We performed comparisons between the clinicopathological variables and miRNAs and we identified miR-7, miR-31, miR-107, miR-124a, miR-183, miR-339, miR-376a and miR-551a as supratentorial-specific (Table 2). We have also identified miR-34a as specifically over-expressed in supratentorial tumors (Table 2). This observation was confirmed using individual assays for miR-34a (data not shown).

Cox regressive analysis was used to select important clinical variables in terms of association with Time to Relapse and Overall Survival. miRNAs were selected by adding each miRNA to the Cox model, one by one. First, using univariate regression analyses we generated a list of statistically significant miRNAs, which were associated with Overall Survival and Time to Relapse (Table 3). Next, we determined the association of these select miRNAs with clinical variables using a multivariate Cox regression analysis. Importantly, miR-203 was identified as an independent factor for Time to Relapse after adjustment for all the known variables (Table 4). These data suggest that miR-203 could be used as a biomarker for management of disease progression in ependymomas as a predictor for the likelihood of recurrence (Table 4). We have also identified three miRNAs (let7d, miR-596 and miR-367) as independent predictors for Overall Survival (Table 4).

**Table 3:** Selected miRNAs after adjusting the clinical variables using Cox regression analyses for both Overall Survival and Time-to-Relapse.

miRNA	Hazard Ratio(95% CI)	Wald-Chisquarestatistics	p-value
**Overall Survival**			
let-7d	1.83(1.11, 3.02)	5.5473	0.0185
miR-596	1.32(1.05, 1.68)	5.3863	0.0203
miR-615	0.74(0.57, 0.96)	4.9653	0.0259
miR-107	1.33(1.03, 1.72)	4.6580	0.0309
miR-302d	0.69(0.48, 0.99)	4.1219	0.0423
miR-193b	0.71(0.50, 1.00)	3.9481	0.0469
miR-497	1.39(1.00, 1.93)	3.9034	0.0482
miR-367	0.75(0.56, 1.00)	3.8851	0.0487
**Time-To-Relapse**			
miR-203	1.35(1.12, 1.63)	9.9520	0.0016
miR-20b	0.65(0.47, 0.92)	5.9878	0.0144
miR-213	0.75(0.59, 0.95)	5.5082	0.0189
miR-432	0.72(0.54, 0.95)	5.3630	0.0206
miR-184	1.20(1.03, 1.41)	5.2786	0.0216
miR-411	0.86(0.75, 0.98)	5.1908	0.0227
miR-376a	0.85(0.73, 0.98)	4.8401	0.0278
miR-381	0.84(0.72, 0.99)	4.6151	0.0317
miR-487b	0.83(0.69, 1.00)	3.8952	0.0484
miR-383	0.87(0.76, 1.00)	3.8949	0.0484

**Table 4:** Multivariate Cox Regression Analyses for Time to Relapse and Overall Survival.

Variable	Hazard Ratio	95% Confidence Interval of Hazard ratio	p-value
**Time-To-Relapse**			
Resection(complete vs. incomplete)	0.17	0.06, 0.48	0.0008
Prior treatment(none vs treated)	0.34	0.14,0.83	0.0175
WHO (II vs III)	0.21	0.076,0.57	0.0024
miR-203 (down vs up)	1.35	1.21,1.63	0.0016
**Overall Survival**			
Resection(complete vs. incomplete)	0.047	0.006, 0.28	0.0007
Let-7d (down×up)	2.78	1.31, 5.89	0.0076
miR-596 (down×up)	1.34	1.01, 1.78	0.0430
miR-367 (up vs down)	0.43	0.25, 0.76	0.0031

**Figure 2:**
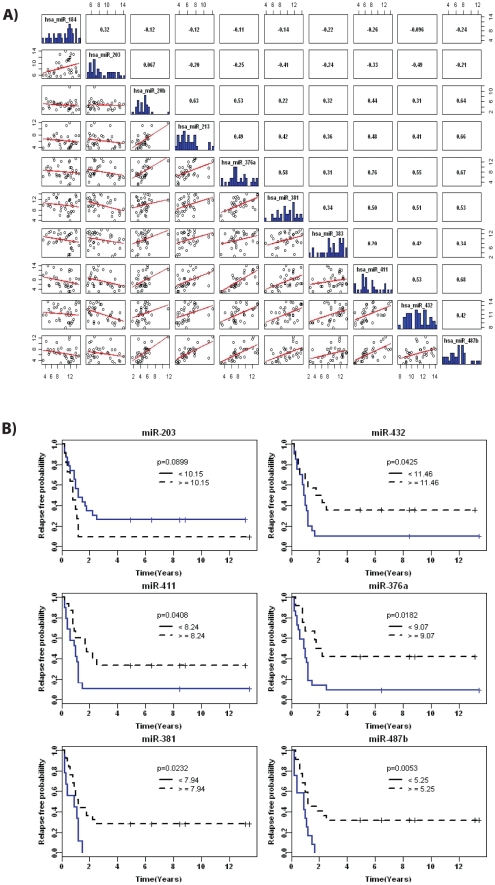
miRNAs and correlations to clinicopathological features. (**A**) Correlation matrix among miRNAs that were identified by Cox regression analyses for Time to Relapse. The lower triangle of the matrix shows the scatter plots of pair-wised relationship between miRNAs. The diagonal panel shows histograms of expression. The upper triangle represents Spearman correlation coefficients for each pair of miRNA (**B**) Kaplan-Meier curves of a cluster of miRNAs on human chromosome 14q32 and associations to Time to Relapse. The thresholds of cut-off points were determined using a survival tree analysis approach. Delta CT values were used for miRNA expression in this analysis. The Y axis represents relapse free probability and the X axis represents time of follow-up in years.

We found that miRNAs whose expression is associated with time to relapse exhibited a strong correlation amongst themselves, thus suggesting that they might be in the same network or pathway (Figure 2A). Five of these (miR- 376a, miR-381, miR-411, miR-432 and miR-487) map to human chromosome 14q32.31. Interestingly, miR-203 also maps to human chromosome 14q32, but to another location (14q32.33).

Finally, we used a survival tree method to determine the thresholds of cut-off points for selected miRNAs based upon their expression levels. We then generated Kaplan-Meier curves for our cohort based upon the expression of the selected miRNAs mapped to human chromosome 14q32. As shown in Figure 2B, all of these miRNAs exhibited statistically significant associations to time to relapse; except miR-203 that showed a trend towards significance. For miR-432, miR-411, miR 376-a, miR-381 and miR-487b, higher expression was associated with a lower relapse free probability. For miR-203, we observed that lower expression correlated to a trend to develop recurrences (Figure 2B). In conclusion, these data demonstrated that a cluster of miRNAs on human chromosome 14q32 might be utilized as biomarkers for this disease.

## Discussion

Ependymoma is the most common pediatric brain tumor with a heterogeneous clinical behavior. Attempts to predict outcomes based on the molecular biology of this tumor have been largely unsuccessful. Our group has already shown clinically relevant epigenomic differences in ependymomas, especially in repetitive regions of the genome [14]. In the present study, we have generated miRNA expression profiles from ependymomas and compared them with those of normal ependymal cells. The normal cells used in this study were derived from normal brain tissue with enrichment for ependymal cells, which originate from radial glial cells, recently suggested as the cell of origin for ependymomas [15]. We have shown that normal controls cluster separately from ependymomas with regard to miRNA expression patterns. Further, miRNA expression profiles were able to differentiate subgroups of tumors from different locations in the brain as shown for protein-coding genes [15], thus reinforcing the idea that ependymomas from different parts of the CNS are distinct entities. For example, Johnson *et al.* have demonstrated that miRNA expression patterns can segregate tumors by location and subgroup [16]. However, our study differs by the methods that were utilized, and in the source of tumor samples. Our group has used Real-Time PCR and paraffin-embedded samples, whereas Johnson *et al.* utilized miRNA microarray hybridization and frozen tumor specimens. Notwithstanding the experimental differences, our findings corroborate theirs in that both groups obtained evidence to support the hypothesis that miRNA profiles are able to subgroup tumors from different locations in the brain.

Studies over the last few years have contributed to our understanding of the genetic abnormalities in ependymomas. Altogether, these investigations have shed some light on the molecular mechanisms underlying the development of ependymomas, while proposing molecular markers of potential value for tumor stratification that might be predictive of clinical behavior. Comparative Genomic hydbirdization (CGH) analysis, for example, have revealed genetic aberrations in ependymomas, such as gains in chromossome 1q that are associated with a poor prognosis [17]. On the other hand, loss of the 6q25.3 region has been associated with improved prognosis [18]. Gene expression analysis have identified molecular signatures in ependymomas that comprise members of both the Notch and Hedgehog signaling pathways [19]. Some studies have also shown that over-expression of the human telomerase (hTERT) is a strong prognostic factor for ependymomas [20]. In addition, ependymomas have shown frequent gain and amplification of the EGFR gene located at 7p11.2 [13], as an independent prognostic factor for poor survival [21]. Several agents targeting EGFR such as erlotinib and gefitinib are currently in clinical trials and the anti-EGFR monoclonal antibody C225 is also being investigated. However, the role of these agents in the treatment of ependymomas remains elusive, hence the urgency to identify better drug targets and molecular biomarkers for this type of brain tumor.

Several lines of evidence have already shown that miRNAs are implicated in the initiation and progression of malignancies. Tumors that have specific miRNAs up- and/or downregulated often exhibit more aggressive phenotypes. Hence, these miRNAs can be exploited as prognostic markers [22]. This is expected considering that a single miRNA can affect an entire network of proteins and/or signaling pathway. In addition, not only may two or more miRNAs act on the same network, they may also co-operatively target a common transcript. These findings suggest that albeit small in size, miRNAs are quite large in their impact to cancer biology.

Initially, we compared the miRNA expression profiles of frozen and paraffin tissue derived from the same patients, and we were able to show a high correlation as previously reported by others [23]. In our study, we were able to identify a miRNA signature that can separate with a 100% certainty normal ependymal cells from ependymomas. Specific miRNAs were confirmed as over-expressed and down-regulated in ependymomas, such as miR-135a, miR-34a, miR-17-5p, miR-383 and miR-485-5p (Figure S1B). We were also able to identify computationally, and by gene expression analysis, some putative targets for these miRNAs (Table S1). Interestingly, we have identified 3 genes from the TGF beta family as putative targets of miR-485-5p. It is noteworthy that the TGF beta family of genes has been previously identified as important in ependymomas and to other brain tumors [24], [25].

A previous study showed that miR-10b was over-expressed in malignant gliomas and in four ependymomas [26]. However, in our study, miR-10b was not differentially expressed, which might be due to sample size differences. On the other hand, we did find miR-10a to be over-expressed in ependymomas in comparison to normal controls (Table 2). This is noteworthy since these miRNAs are part of the same miRNA family. Differential expression of other miRNAs was associated to ependymomas [27]. This group used a small sample size and miRNA expression profiling was evaluated by microarray hybridization [27]. miR-21 was also identified as being over-expressed in ependymomas [27]. Other groups have already shown increased expression of miR-21 in brain tumors [28], [29] and we have demonstrated that this miR is over-expressed in our samples.

We also report differential miRNA expression and its association with clinicopathologic features in our patient cohort. We have found concerted differential expression of miRNAs that are organized in clusters in the genome. Accordingly, miRNAs in the miR-17-92 genomic cluster, such as miR-17-5p, miR-19a, miR-19b and miR-20a, were up-regulated in ependymomas (Table 2). We have also observed site-specific expression for miR-7, miR-31, miR-107, miR-124, miR-183, miR-339, miR-376a and miR-551a, when comparing supratentorial to infratentorial tumors (Table 2). Using individual assays, we were able to confirm that miR-34a is highly over-expressed in supratentorial tumors relative to normal supratentorial controls. It is known that supratentorial ependymomas tend to have a better prognosis. This was indeed the case in our patient cohort: 2 out of 6 (33%) patients with supratentorial ependymoma succumbed to the disease, in contrast to 15 out of 20 (75%) with an infratentorial tumor (Figure S2A). Future studies will determine whether this observed altered miRNA expression has significance to the differential clinical behavior of supratentorial and infratentorial ependymomas.

We have applied Cox Regression analyses to identify miRNAs with differential expression associated to both overall survival and time to relapse (Table 3). Importantly, we have also demonstrated significant concerted dysregulation of expression among the miRNAs identified in the Cox Regression analyses (Figure 2A). Based on these results, we hypothesized that some of these miRNAs might be in the same genomic location, or under the same regulatory control. Interestingly, 6 miRNAs identified in our analyses are located in the same human chromosome, and five of them are in the same genomic cluster. This cluster is mapped to human chromosome 14q32.31, which is well known for the presence of imprinted genes. We have further shown that miRNAs from this imprinted cluster are associated with time to relapse in our ependymoma cohort by Kaplan-Meier curves (Figure 2B). This cluster of miRNAs is mapped to a genomic region that is regulated by DNA methylation in both humans and mice [30], and miRNAs from this genomic cluster have already been associated to human malignancies [31]. This indicates that human chromosome 14q32.31 may be implicated in the tumorigenesis of ependymomas. Our findings highlight, for the first time, a strong association of this imprinted cluster of miRNAs on 14q32.31 to the development of ependymomas. These findings may direct future investigations aimed at the development of new drugs and therapies for ependymomas. However, more research is needed to better understand the function of these miRNAs and the expression of other genes mapped to the same genomic region.

Next, we performed multivariate analyses to determine if any of these miRNAs could be of value as a biomarker for ependymomas. Using this approach, we have shown that miRNA-203 down-regulation is strongly associated with Time to Relapse (Table 4 and Figure 2A). We found it to be an independent prognostic factor for recurrences with a hazard ratio that is almost ten times higher than that of other known clinical features such as extent of resection (Table 4). MiR-203 is also mapped to human chromosome 14, however it is located at a distance of approximately 3 megabases from the aforementioned imprinted cluster of miRNAs identified in our univariate analyses. Importantly, previous studies have shown the association of miR-203 to human cancers. For example, epigenetic silencing of miR-203 has been implicated in the regulation of the BCR-ABL gene in leukemia [32]. This miRNA has also been associated to skin differentiation by repressing cell “stemness” [33]. Moreover, it was recently shown that miR-203 expression in keratinocytes is dependent on the regulation of the levels of the tumor suppressor gene p53 [34]. MiR-203 expression was also associated to a worse survival of patients with pancreatic tumors [35]. Finally, aberrant expression of miR-203 was correlated to different clinicopathologic variables in gastric and colorectal cancers [36]. Multivariate analyses for Overall Survival also revealed that a member of the let-7 miRNA family, let-7d, is a prognostic marker in ependymomas (Table 4). The let-7 family has been extensively associated with embryogenesis, pluripotency and cancers [37] Additionally, miR-596 and miR-367 were also associated to Overall Survival (Table 4). A few reports have implicated both miRNAs with tumorigenesis and embryogenesis [38], [39].

Notwithstanding the relatively small size of our patient cohort, we were able to identify miRNAs of clinical importance. Our results indicate, for the first time, that miRNAs might prove invaluable as a biomarker for recurrence in ependymomas. Additionally, we are further evaluating the molecular mechanisms underlying the observed differential expression of miRNAs with clinical significance. In conclusion, we were able to identify a ependymoma-specific miRNA signature, a tumor site-specific miRNA profile that enables stratification of intracranial ependymomas according to region of occurrence (infratentorial or supratentorial), and altered miRNA expression associated to certain clinical behavior. We have identified a miRNA cluster in an imprinted region of human chromosome 14q32 that is associated to a worse prognosis in ependymomas. Specifically, miR-203 was identified as an independent prognostic marker for time to relapse compared to the clinical variables that are presently utilized in the clinics. Moreover, let-7d, miR-596 and miR-367 were strongly associated to Overall Survival. We are currently working to confirm some of the predicted targets for these miRNAs. We believe that these findings will pave new ways for the development of therapies and for a better management of this type of pediatric brain tumor.

## Supporting Information

Figure S1 Supervised hierarchical clustering and Individual confirmation for 4 differentially expressed miRNAs in ependymomas. (**A**) Supervised Hierarchical Clustering with twenty-eight miRNAs identified as differentially expressed in ependymomas when compared to normal controls (p≤0.01,FDR<0.05). The clustering and tree are based on Pearson's correlation and were generated by the MeV 4.1 software according to Delta Ct values. (**B**) Histograms for individual assays were generated by the StatMiner software and represent fold change differences using individual TaqMan Real-Time PCR assays for normal controls and ependymoma samples (p≤0.05). Over-expressed miRs (miR-135a and miR-17-5p) and down-regulated miRNAs (miR-383 and miR-485-5p) are represented.
(AI) Click here for additional data file.

Figure S2 Kaplan-Meier curves for Overall Survival (OS) based on the Log Rank Test using all the samples in this study for clinicopathologic variables. (**A**) Tumor Location, (**B**) Resection and (**C**) Tumor Specimen.
(AI) Click here for additional data file.

Figure S3 Kaplan-Meier curves for Time to Relapse based on the Log Rank Test using all the samples in this study for clinicopathologic variables. (**A**) Resection, (**B**) Prior treatment.
(AI) Click here for additional data file.

Table S1 List of microRNAs present in the TLDA array v1.0 that were used in this study.
(PDF)

Table S2 Target identification for differentially expressed miRNAs in ependymomas based on GoMir, GeneGo, literature search and microarray gene expression analyses (p<0.05).
(DOC)
