# Supplementary material for: Identification of MicroRNAs as Potential Prognostic Markers in Ependymoma
Source: PLoS One. 2011 Oct 28;6(10):e25114. doi: 10.1371/journal.pone.0025114 (PMC3203863; doi:10.1371/journal.pone.0025114)
Supplement: Table S1 — List of microRNAs present in the TLDA array v1.0 that were used in this study. (PDF) [file pone.0025114.s004.pdf]

Table S1: List of microRNAs present in the TLDA array v1.0 that were used in this study.
Generated from AIF v5, 01/05/2007
Note: For controls, the miRBase names, accessions, and aliases come from NCBI.

Part Number	AB Assay ID	Target Sequence	Assay Name	miRBase ID	miRBase Accessions	miRBase Alias	Control	Homo sapiens	Mus musculus	Rattus norvegicus	Arabidopsis thaliana	Caenorhabditis elegans	Drosophila melanogaster
4373169	000377	UGAGGUAGUAGGUUGUAUAGUU	hsa-let-7a	bta-let-7a::cbr-let-7::	MIMAT000(	gga-let-7a-1(5.0)::gga-le		+		+		+	
4373168	000378	UGAGGUAGUAGGUUGUGUGGUU	hsa-let-7b	dre-let-7b::fru-let-7b:	MIMAT0000063::MIMAT0000522::M			+	+	+			
4373167	000379	UGAGGUAGUAGGUUGUAUGGUU	hsa-let-7c	dre-let-7c::gga-let-7(	MIMAT0000064::MIMAT0000523::M			+	+	+			
4373166	000380	AGAGGUAGUAGGUUGCAUAGU	hsa-let-7d	hsa-let-7d::mdo-let-7	MIMAT0000065::MIMAT0000383::M			+	+	+			
4373165	000381	UGAGGUAGGAGGUUGUAUAGU	hsa-let-7e	hsa-let-7e::mmu-let-	MIMAT0000066::MIMAT0000524::M			+	+	+			
4373163	000383	UGAGGUAGUAGUUUGUACAGU	hsa-let-7g	gga-let-7g::hsa-let-7	MIMAT0000121::MIMAT0000414::M			+	+				
4373153	000387	UACCCUGUAGAUCCGAAUUUGUG	hsa-miR-10a	age-miR-10a::bta-m	MIMAT000(	dre-miR-10a(6.0)		+	+	+			
4373152	000388	UACCCUGUAGAACCGAAUUUGU	hsa-miR-10b	gga-miR-10b::ggo-m	MIMAT000(	dre-miR-10b(6.0)::rno-m		+					
4373120	000392	ACUGCAGUGAAGGCACUUGU	hsa-miR-17-3p	age-miR-17-3p::bta-	MIMAT0000071::MIMAT0001115::MI			+					
4373119	000393	CAAAGUGCUUACAGUGCAGGUAGU	hsa-miR-17-5p	age-miR-17-5p::bta-	MIMAT0000070::MIMAT0000649::M			+	+	+			
4373118	000394	UAAGGUGCAUCUAGUGCAGAUA	hsa-miR-18a	age-miR-18::bta-miR	MIMAT000(	hsa-miR-18(6.0)		+	+	+			
4373099	000395	UGUGCAAAUCUAUGCAAAACUGA	hsa-miR-19a	age-miR-19a::dre-m	MIMAT0000073::MIMAT0000651::M			+	+	+			
4373098	000396	UGUGCAAAUCCAUGCAAAACUGA	hsa-miR-19b	age-miR-19b::dre-m	MIMAT0000074::MIMAT0000513::M			+	+	+			
4373090	000397	UAGCUUAUCAGACUGAUGUUGA	hsa-miR-21	age-miR-21::gga-mi	MIMAT0000076::MIMAT0000530::M			+	+	+			
4373072	000402	UGGCUCAGUUCAGCAGGAACAG	hsa-miR-24	bta-miR-24::dre-miR	MIMAT0000080::MIMAT0000219::M			+	+	+			
4373278	000425	UGGCAGUGUCUUAGCUGGUUGUU	hsa-miR-34a	gga-miR-34a::hsa-m	MIMAT000(	dre-miR-34a(6.0)		+	+	+			
4373037	000427	UAGGCAGUGUCAUUAGCUGAUUG	hsa-miR-34b	hsa-miR-34b	MIMAT0000685			+					
4373036	000428	AGGCAGUGUAGUUAGCUGAUUGC	hsa-miR-34c	gga-miR-34c::hsa-m	MIMAT0000381::MIMAT0000686::M			+	+	+			
4373013	000430	UAUUGCACUUGUCCCGGCCUG	hsa-miR-92	gga-miR-92::hsa-mi	MIMAT0000092::MIMAT0000539::M			+	+	+			
4373012	000432	AAAGUGCUGUUCGUGCAGGUAG	hsa-miR-93	age-miR-93::ggo-mi	MIMAT0000093::MIMAT0002759::M			+					
4373154	000443	AGCAGCAUUGUACAGGGCUAUCA	hsa-miR-107	dre-miR-107::fru-miR	MIMAT0000104::MIMAT0000647::M			+	+	+			
4373137	000463	UAACACUGUCUGGUAAAGAUGG	hsa-miR-141	hsa-miR-141::mmu-i	MIMAT0000153::MIMAT0000432::M			+	+	+			
4373136	000464	UGUAGUGUUUCCUACUUUAUGGA	hsa-miR-142-3p	dre-miR-142a-3p::hs	MIMAT000(	gga-miR-142-3p(8.1)		+		+			
4373135	000465	CAUAAAGUAGAAAGCACUAC	hsa-miR-142-5p	bta-miR-142::hsa-mi	MIMAT000(	gga-miR-142-5p(8.1)		+	+	+			
4373132	000468	UGAGAACUGAAUUCCAUGGGUU	hsa-miR-146a	gga-miR-146a::hsa-i	MIMAT000(	gga-miR-146(7.1)::hsa-r		+	+	+			
4373124	000479	UUAAUGCUAAUCGUGAUAGGGG	hsa-miR-155	dre-miR-155::gga-m	MIMAT0000646::MIMAT0001106::M			+					
4373115	000482	AACAUUCAACCUGUCGGUGAGU	hsa-miR-181c	ggo-miR-181c::hsa-i	MIMAT0000258::MIMAT0000674::M			+	+	+			
4373109	000490	CAACGGAAUCCCAAAAGCAGCU	hsa-miR-191	hsa-miR-191::mdo-n	MIMAT0000221::MIMAT0000440::M			+	+	+			
4373096	000505	UAAUACUGCCGGGUAAUGAUGG	hsa-miR-200c	hsa-miR-200c::mdo-	MIMAT0000617::MIMAT0000657::M			+	+	+			
4373084	000518	AUGACCUAUGAAUUGACAGAC	hsa-miR-215	gga-miR-215::ggo-m	MIMAT0000272::MIMAT0001134::M			+					
4373081	000521	UUGUGCUUGAUCUAACCAUGU	hsa-miR-218	age-miR-218::bta-m	MIMAT000(	gga-miR-218-1(5.0)::gga		+	+	+			
4373077	000524	AGCUACAUUGUCUGCUGGGUUUC	hsa-miR-221	dre-miR-221::fru-miR	MIMAT0000278::MIMAT0000890::M			+		+			
4373075	000526	UGUCAGUUUGUCAAAUACCCC	hsa-miR-223	dre-miR-223::fru-miR	MIMAT0000280::MIMAT0000665::M			+	+	+			
4373064	000528	CAGUGCAAUAGUAUUGUCAAAGC	hsa-miR-301	hsa-miR-301::mmu-i	MIMAT0000379::MIMAT0000688::M			+	+				
4373039	000552	UGCUGACUCCUAGUCCAGGGC	hsa-miR-345	hsa-miR-345	MIMAT0000772			+					
4373029	000560	AAAGUGCUGCGACAUUUGAGCGU	hsa-miR-372	hsa-miR-372	MIMAT0000724			+					
4373027	000564	UUUGUUCGUUCGGCUCGCGUGA	hsa-miR-375	hsa-miR-375::mmu-i	MIMAT0000728::MIMAT0000739			+	+				
4373024	000567	CUCCUGACUCCAGGUCCUGUGU	hsa-miR-378	hsa-miR-378::mmu-i	MIMAT0000731::MIMAT0000742::M			+	+	+			
4373286	000580	UAAAGUGCUUAUAGUGCAGGUAG	hsa-miR-20a	bta-miR-20a::dre-mi	MIMAT000(	gga-miR-20(5.1)::hsa-m		+	+	+			
4373285	000583	UCUUUGGUUAUCUAGCUGUAUGA	hsa-miR-9	aga-miR-9a::age-mi	MIMAT000(	mmu-miR-9(5.0)		+		+			+
4373174	000593	UAUUGCUUAAGAAUACGCGUAG	hsa-miR-137	gga-miR-137::hsa-m	MIMAT000(	mmu-miR-137(5.0)		+		+			
4373273	001011	CAUCUUACCGGACAGUGCUGGA	hsa-miR-200a#	hsa-miR-200a*	MIMAT0001620			+					
4373263	001014	CAAAGUGCUCAUAGUGCAGGUAG	hsa-miR-20b	gga-miR-20b::hsa-m	MIMAT0001348::MIMAT0001411::M			+					
4373178	001097	UGAGAACUGAAUUCCAUAGGCU	hsa-miR-146b	hsa-miR-146b::mmu	MIMAT0002809::MIMAT0003475			+	+				
4373116	001098	AACAUUCAUUGCUGUCGGUGGG	hsa-miR-181b	dre-miR-181b::fru-m	MIMAT0000257::MIMAT0000673::M			+	+	+			
4373180	001099	AACAUUCAUUGUUGUCGGUGGGUU	hsa-miR-181d	hsa-miR-181d	MIMAT0002821			+					
4373161	000385	UGGAAUGUAAAGAAGUAUGUA	hsa-miR-1	cbr-miR-1::cel-miR-1	MIMAT000(	gga-miR-1(7.1)::gga-miR		+	+			+	
4373070	000404	UUCAAGUAAUCCAGGAUAGGC	hsa-miR-26a	gga-miR-26a::hsa-m	MIMAT0000082::MIMAT0000533::M			+	+	+			
4373069	000406	UUCAAGUAAUUCAGGAUAGGUU	hsa-miR-26b	bta-miR-26b::hsa-mi	MIMAT0000083::MIMAT0000534::M			+	+	+			
4373287	000408	UUCACAGUGGCUAAGUUCCGC	hsa-miR-27a	hsa-miR-27a::mdo-n	MIMAT0000084::MIMAT0000537::M			+	+	+			
4373068	000409	UUCACAGUGGCUAAGUUCUGC	hsa-miR-27b	bta-miR-27b::gga-m	MIMAT0000126::MIMAT0000419::M			+	+	+			
4373158	000439	AGCAGCAUUGUACAGGGCUAUGA	hsa-miR-103	age-miR-103::bta-m	MIMAT000(	gga-miR-103-1(5.0)::gga		+	+	+			
4373149	000448	UCCCUGAGACCCUUUAACCUGUG	hsa-miR-125a	bta-miR-125a::hsa-n	MIMAT0000135::MIMAT0000443::M			+	+	+			
4373148	000449	UCCCUGAGACCCUAACUUGUGA	hsa-miR-125b	aga-miR-125::age-m	MIMAT0000136::MIMAT0000397::M			+	+	+			+
4373126	000474	UCAGUGCAUGACAGAACUUGGG	hsa-miR-152	hsa-miR-152::mdo-n	MIMAT0000162::MIMAT0000438::M			+	+	+			
4373114	000484	UAUGGCACUGGUAGAAUUCACUG	hsa-miR-183	dre-miR-183::fru-miR	MIMAT0000212::MIMAT0000261::M			+	+	+			
4373089	000512	CUGUGCGUGUGACAGCGGCUGA	hsa-miR-210	hsa-miR-210::mmu-i	MIMAT0000267::MIMAT0000658::M			+	+	+			
4373076	000525	AGCUACAUCUGGCUACUGGGUCUC	hsa-miR-222	age-miR-222::dre-m	MIMAT0000279::MIMAT0000670::M			+	+	+			
4373066	000527	AGGGCCCCCCCUCAAUCCUGU	hsa-miR-296	hsa-miR-296::mmu-i	MIMAT0000374::MIMAT0000690::M			+	+	+			
4373275	000530	UAAACGUGGAUGUACUUGCUUU	hsa-miR-302a#	hsa-miR-302a*	MIMAT0000683			+					
4373277	000534	UUUAACAUGGGGGUACCUGCUG	hsa-miR-302c#	hsa-miR-302c*	MIMAT0000716			+					
4373063	000535	UAAGUGCUUCCAUGUUUGAGUGU	hsa-miR-302d	hsa-miR-302d	MIMAT000(	mmu-miR-302d(8.1)		+					
4373052	000539	CGCAUCCCCUAGGGCAUUGGUGU	hsa-miR-324-5p	hsa-miR-324-5p::rnc	MIMAT000(	mmu-miR-324-5p(5.0)		+		+			
4373034	000555	AAUUGCACUUUAGCAAUGGUGA	hsa-miR-367	hsa-miR-367	MIMAT0000719			+					
4373015	000576	AGCUCGGUCUGAGGCCCCUCAG	hsa-miR-423	hsa-miR-423::mmu-i	MIMAT0001340::MIMAT0003454			+	+				
4373053	000579	CCACUGCCCCAGGUGCUGCUGG	hsa-miR-324-3p	hsa-miR-324-3p::mn	MIMAT0000554::MIMAT0000556::M			+	+	+			
4373195	001021	AGAUCGACCGUGUUAUAUUCGC	hsa-miR-369-5p	hsa-miR-369-5p	MIMAT0001621			+					
4373207	001030	UGGCAGUGUAUUGUUAGCUGGU	hsa-miR-449	cfa-miR-449::hsa-mi	MIMAT0001541::MIMAT0001542::M			+	+	+			
4373222	001043	CAGCAGCACACUGUGGUUUGU	hsa-miR-497	hsa-miR-497	MIMAT0002820			+					
4373226	001047	AAUCCUUUGUCCCUGGGUGAGA	hsa-miR-501	hsa-miR-501	MIMAT0002872			+					
4373234	001053	UGAUUGGUACGUCUGUGGGUAGA	hsa-miR-509	hsa-miR-509	MIMAT0002881			+					
4373236	001111	GUGUCUUUUGCUCUGCAGUCA	hsa-miR-511	hsa-miR-511	MIMAT0002808			+					
4373242	001112	UUCUCCAAAAGAAAGCACUUUCUG	hsa-miR-515-5p	hsa-miR-515-5p	MIMAT0002826			+					
4373252	001116	AAAGUGCUUCCUUUUAGAGGG	hsa-miR-520b	hsa-miR-520b	MIMAT0002843			+					
4373253	001117	AAAGUGCUUCCUUUUAGAGGGUU	hsa-miR-520c	hsa-miR-520c	MIMAT0002846			+					
4373254	001118	AAAGUGCUUCUCUUUGGUGGGUU	hsa-miR-520d	hsa-miR-520d	MIMAT0002856			+					
4373255	001119	AAAGUGCUUCCUUUUUGAGGG	hsa-miR-520e	hsa-miR-520e	MIMAT0002825			+					
4373256	001120	AAGUGCUUCCUUUUAGAGGGUU	hsa-miR-520f	hsa-miR-520f	MIMAT0002830			+					
4373257	001121	ACAAAGUGCUUCCCUUUAGAGUGU	hsa-miR-520g	hsa-miR-520g	MIMAT0002858			+					
4373235	001143	UACUCAGGAGAGUGGCAAUCACA	hsa-miR-510	hsa-miR-510	MIMAT0002882			+					
4373240	001147	AUUGACACUUCUGUGAGUAG	hsa-miR-514	hsa-miR-514	MIMAT0002883			+					
4373241	001148	GAGUGCCUUCUUUUGGAGCGU	hsa-miR-515-3p	hsa-miR-515-3p	MIMAT0002827			+					
4373243	001151	AUCGUGCAUCCCUUUAGAGUGUU	hsa-miR-517a	hsa-miR-517a	MIMAT0002852			+					
4373244	001152	UCGUGCAUCCCUUUAGAGUGUU	hsa-miR-517b	hsa-miR-517b	MIMAT0002857			+					
4373264	001153	AUCGUGCAUCCUUUUAGAGUGU	hsa-miR-517c	hsa-miR-517c	MIMAT0002866			+					
4373186	001154	AAAGCGCUUCCCUUUGCUGGA	hsa-miR-518a	hsa-miR-518a	MIMAT0002863			+					
4373246	001156	CAAAGCGCUCCCCUUUAGAGGU	hsa-miR-518b	hsa-miR-518b	MIMAT0002844			+					
4373247	001157	CAAAGCGCUUCUCUUUAGAGUG	hsa-miR-518c	hsa-miR-518c	MIMAT0002848			+					
4373248	001159	CAAAGCGCUUCCCUUUGGAGC	hsa-miR-518d	hsa-miR-518d	MIMAT0002864			+					
4373265	001160	AAAGCGCUUCCCUUCAGAGUGU	hsa-miR-518e	hsa-miR-518e	MIMAT0002861			+					
4373268	001167	AAAGUGCUUCCCUUUGGACUGU	hsa-miR-520a	hsa-miR-520a	MIMAT0002834			+					
4373258	001170	ACAAAGUGCUUCCCUUUAGAGU	hsa-miR-520h	hsa-miR-520h	MIMAT0002867			+					
4373062	000416	CUUUCAGUCGGAUGUUUGCAGC	hsa-miR-30a-3p	dre-miR-30e*::gga-n	MIMAT000(	hsa-miR-30a(3.1)::mmu		+	+	+			
4373061	000417	UGUAAACAUCCUCGACUGGAAG	hsa-miR-30a-5p	gga-miR-30a-5p::gg	MIMAT0000087::MIMAT0000128::M			+	+	+			
4373060	000419	UGUAAACAUCCUACACUCUCAGC	hsa-miR-30c	bta-miR-30c::hsa-mi	MIMAT000(	gga-miR-30c(8.1)::gga-r		+	+	+			

4373059	000420	UGUAAACAUCCCCGACUGGAAG	hsa-miR-30d	dre-miR-30d::fru-miF	MIMAT0000245::MIMAT0000515::M			+	+	+	
4373057	000422	CUUUCAGUCGGAUGUUUACAGC	hsa-miR-30e-3p	hsa-miR-30e-3p	MIMAT0000693			+			
4373011	000433	UUCAACGGGUAUUUAUUGAGCA	hsa-miR-95	ggo-miR-95::hsa-mil	MIMAT0000094::MIMAT0002142::M			+			
4373160	000437	AACCCGUAGAUCCGAACUUGUG	hsa-miR-100	aga-miR-100::age-m	MIMAT0000098::MIMAT0000655::M			+	+	+	
4373159	000438	UACAGUACUGUGAUAACUGAAG	hsa-miR-101	age-miR-101::dre-m	MIMAT000(	rno-miR-101(7.1)		+	+	+	
4373269	000451	CAUUAUUACUUUUGGUACGCG	hsa-miR-126#	dre-miR-126*::gga-n	MIMAT000(	mmu-miR-126*(5.0)		+	+	+	
4373147	000452	UCGGAUCCGUCUGAGCUUGGCU	hsa-miR-127	age-miR-127::bta-m	MIMAT000(	mmu-miR-127(5.0)		+		+	
4373143	000457	UAACAGUCUACAGCCAUGGUCG	hsa-miR-132	bta-miR-132::dre-mi	MIMAT0000144::MIMAT0000426::M			+	+	+	
4373142	000458	UUGGUCCCCUUCAACCAGCUGU	hsa-miR-133a	aga-miR-133::age-m	MIMAT000(	mmu-miR-133(3.0)		+	+	+	+
4373140	000460	UAUGGCUUUUUAUUCCUAUGUGA	hsa-miR-135a	age-miR-135::dre-m	MIMAT000(	gga-miR-135a-1(5.0)::gç		+	+	+	
4373139	000461	UAUGGCUUUUCAUUCCUAUGUG	hsa-miR-135b	hsa-miR-135b::mdo-	MIMAT0000611::MIMAT0000612::M			+	+	+	
4373130	000470	UCAGUGCACUACAGAACUUUGU	hsa-miR-148a	bta-miR-148a::gga-r	MIMAT000(	hsa-miR-148(3.0)		+	+		
4373129	000471	UCAGUGCAUCACAGAACUUUGU	hsa-miR-148b	bta-miR-148b::hsa-n	MIMAT0000579::MIMAT0000580::M			+	+	+	
4373113	000485	UGGACGGAGAACUGAUAAGGGU	hsa-miR-184	gga-miR-184::hsa-m	MIMAT0000213::MIMAT0000454::M			+	+	+	
4373108	000491	CUGACCUAUGAAUUGACAGCC	hsa-miR-192	hsa-miR-192::rno-m	MIMAT0000222::MIMAT0000867			+		+	
4373106	000493	UGUAACAGCAACUCCAUGUGGA	hsa-miR-194	age-miR-194::gga-m	MIMAT0000224::MIMAT0000460::M			+	+	+	
4373095	000507	GUGAAAUGUUUAGGACCACUAG	hsa-miR-203	hsa-miR-203::rno-m	MIMAT0000264::MIMAT0000876			+		+	
4373094	000508	UUCCCUUUGUCAUCCUAUGCCU	hsa-miR-204	dre-miR-204::fru-miF	MIMAT000(	fru-miR-204a(8.0)::gga-i		+		+	
4373092	000510	UGGAAUGUAAGGAAGUGUGUGG	hsa-miR-206	dre-miR-206::gga-m	MIMAT0000239::MIMAT0000462::M			+	+	+	
4373088	000514	UUCCCUUUGUCAUCCUUCGCCU	hsa-miR-211	hsa-miR-211::mne-n	MIMAT0000268::MIMAT0002548::M			+			
4373083	000519	UAAUCUCAGCUGGCAACUGUG	hsa-miR-216	gga-miR-216::ggo-m	MIMAT000(	dre-miR-216(6.0)::mmu-		+	+	+	
4373082	000520	UACUGCAUCAGGAACUGAUUGGAU	hsa-miR-217	gga-miR-217::ggo-m	MIMAT000(	dre-miR-217(6.0)		+			
4373047	000544	GCAAAGCACACGGCCUGCAGAGA	hsa-miR-330	hsa-miR-330	MIMAT0000751			+			
4373045	000546	UCAAGAGCAAUAACGAAAAAUGU	hsa-miR-335	hsa-miR-335::mmu-i	MIMAT0000575::MIMAT0000765::M			+	+	+	
4373030	000559	GUGCCGCCAUCUUUUGAGUGU	hsa-miR-371	hsa-miR-371	MIMAT0000723			+			
4373023	000568	UGGUAGACUAUGGAACGUA	hsa-miR-379	hsa-miR-379::rno-m	MIMAT0000733::MIMAT0003192			+		+	
4373020	000571	UAUACAAGGGCAAGCUCUCUGU	hsa-miR-381	hsa-miR-381::mmu-i	MIMAT0000736::MIMAT0000746			+	+		
4373018	000573	AGAUCAGAAGGUGAUUGUGGCU	hsa-miR-383	gga-miR-383::hsa-m	MIMAT0000738::MIMAT0003363::M			+			
4373101	000581	GGUCCAGAGGGGAGAUAGG	hsa-miR-198	ggo-miR-198::hsa-m	MIMAT0000228::MIMAT0002605::M			+			
4373172	000591	UUGGUCCCCUUCAACCAGCUA	hsa-miR-133b	gga-miR-133b::hsa-i	MIMAT0000769::MIMAT0000770::M			+	+	+	
4373187	000599	CAAGUCACUAGUGGUUCCGUUUA	hsa-miR-224	ggo-miR-224::hsa-m	MIMAT0000281::MIMAT0002132::M			+		+	
4373188	000600	UGGUUUACCGUCCCACAUACAU	hsa-miR-299-5p	hsa-miR-299-5p::mr	MIMAT000(	hsa-miR-299(6.0)		+	+	+	
4373290	000602	UGUAAACAUCCUACACUCAGCU	hsa-miR-30b	bta-miR-30b::dre-mi	MIMAT0000130::MIMAT0000420::M			+	+	+	
4373197	001022	AGGUUACCCGAGCAACUUUGCA	hsa-miR-409-5p	hsa-miR-409-5p	MIMAT0001638			+			
4373280	001026	UCUUGGAGUAGGUCAUUGGGUGG	hsa-miR-432	hsa-miR-432::oar-m	MIMAT0001416::MIMAT0002814			+			
4373205	001028	AUCAUGAUGGGCUCCUCGGUGU	hsa-miR-433	hsa-miR-433::mmu-i	MIMAT0001420::MIMAT0001627::M			+	+	+	
4373212	001036	AGAGGCUGGCCGUGAUGAAUUC	hsa-miR-485-5p	hsa-miR-485-5p::mr	MIMAT0002175::MIMAT0003128::M			+	+	+	
4373219	001041	UGAAACAUACACGGGAAACCUCUU	hsa-miR-494	hsa-miR-494	MIMAT0002816			+			
4373231	001050	UAAGGCACCCUUCUGAGUAGA	hsa-miR-506	hsa-miR-506	MIMAT0002878			+			
4373233	001052	UGAUUGUAGCCUUUUGGAGUAGA	hsa-miR-508	hsa-miR-508	MIMAT0002880			+			
4373190	001100	GGCAAGAUGCUGGCAUAGCUG	hsa-miR-31	ggo-miR-31::hsa-mil	MIMAT0000089::MIMAT0002379::M			+			
4373214	001107	AGUGACAUCACAUAUACGGCAGC	hsa-miR-489	hsa-miR-489	MIMAT0002805			+			
4373259	001122	AACGCACUUCCCUUUAGAGUGU	hsa-miR-521	hsa-miR-521	MIMAT0002854			+			
4373014	000386	UGGAAGACUAGUGAUUUUGUUG	hsa-miR-7	gga-miR-7::hsa-miR	MIMAT000(	dre-miR-7(6.0)		+	+		
4373073	000400	AUCACAUUGCCAGGGAUUACC	hsa-miR-23b	gga-miR-23b::hsa-m	MIMAT0000125::MIMAT0000418::M			+	+	+	
4373067	000411	AAGGAGCUCACAGUCUAUUGAG	hsa-miR-28	age-miR-28::ggo-mil	MIMAT0000085::MIMAT0000653::M			+	+	+	
4373056	000423	UAUUGCACAUUACUAAGUUGC	hsa-miR-32	gga-miR-32::ggo-mil	MIMAT0000090::MIMAT0000654::M			+	+	+	
4373141	000459	UGUGACUGGUUGACCAGAGGG	hsa-miR-134	ggo-miR-134::hsa-m	MIMAT000(	mmu-miR-134(5.0)		+		+	
4373131	000469	GUGUGUGGAAAUGCUUCUGC	hsa-miR-147	hsa-miR-147::mne-n	MIMAT0000251::MIMAT0002474::M			+			
4373128	000472	UCUGGCUCCGUGUCUUCACUCC	hsa-miR-149	hsa-miR-149::mmu-i	MIMAT0000159::MIMAT0000450			+	+		
4373125	000476	UUGCAUAGUCACAAAAGUGA	hsa-miR-153	gga-miR-153::ggo-m	MIMAT000(	mmu-miR-153(5.0)		+		+	
4373112	000486	CAAAGAAUUCUCCUUUUGGGCUU	hsa-miR-186	ggo-miR-186::hsa-m	MIMAT0000215::MIMAT0000456::M			+	+	+	
4373111	000487	UCGUGUCUUGUGUUGCAGCCG	hsa-miR-187	ggo-miR-187::hsa-m	MIMAT0000262::MIMAT0000864::M			+		+	
4373110	000489	UGAUAUGUUUGAUAUAUUAGGU	hsa-miR-190	dre-miR-190::fru-miF	MIMAT0000220::MIMAT0000458::M			+	+	+	
4373107	000492	AACUGGCCUACAAAGUCCCAG	hsa-miR-193a	hsa-miR-193a::mdo-	MIMAT000(	hsa-miR-193(6.0)		+	+	+	
4373104	000495	UAGGUAGUUUCAUGUUGUUGG	hsa-miR-196a	gga-miR-196::ggo-m	MIMAT000(	dre-miR-196a(6.0)::gga-		+	+	+	
4373103	000496	UAGGUAGUUUCCUGUUGUUGG	hsa-miR-196b	hsa-miR-196b::mdo-	MIMAT0001080::MIMAT0001081::M			+	+	+	
4373102	000497	UUCACCACCUUCUCCACCCAGC	hsa-miR-197	age-miR-197::hsa-m	MIMAT0000227::MIMAT0002600::M			+			
4373093	000509	UCCUUCAUUCCACCGGAGUCUG	hsa-miR-205	age-miR-205::bta-m	MIMAT0000238::MIMAT0000266::M			+	+	+	
4373091	000511	AUAAGACGAGCAAAAAGCUUGU	hsa-miR-208	hsa-miR-208::mmu-i	MIMAT0000241::MIMAT0000520::M			+	+	+	
4373086	000516	ACCAUCGACCGUUGAUUGUACC	hsa-miR-213	dre-miR-181a*::fru-r	MIMAT000(	fru-miR-213(8.0)::gga-m		+	+	+	
4373085	000517	ACAGCAGGCACAGACAGGCAG	hsa-miR-214	age-miR-214::dre-m	MIMAT0000271::MIMAT0000661::M			+	+	+	
4373078	000523	CCACACCGUAUCUGACACUUU	hsa-miR-220	hsa-miR-220::ppa-m	MIMAT0000277::MIMAT0002579::M			+			
4373055	000536	AAAAGCUGGGUUGAGAGGGCGAA	hsa-miR-320	hsa-miR-320::mmu-i	MIMAT0000510::MIMAT0000666::M			+	+	+	
4373051	000540	CCUAGUAGGUGUCCAGUAAGUGU	hsa-miR-325	hsa-miR-325	MIMAT0000771			+			
4373050	000542	CCUCUGGGCCCUUCCUCCAG	hsa-miR-326	hsa-miR-326::ssc-m	MIMAT0000756::MIMAT0002140			+			
4373049	000543	CUGGCCCUCUCUGCCCUUCCGU	hsa-miR-328	hsa-miR-328::mmu-i	MIMAT0000564::MIMAT0000565::M			+	+	+	
4373046	000545	GCCCCUGGGCCUAUCCUAGAA	hsa-miR-331	hsa-miR-331::mmu-i	MIMAT0000570::MIMAT0000571::M			+	+	+	
4373044	000547	UCCAGCUCCUAUAUGAUGCCUUU	hsa-miR-337	hsa-miR-337	MIMAT0000754			+			
4373042	000549	UCCCUGUCCUCCAGGAGCUCA	hsa-miR-339	hsa-miR-339::mmu-i	MIMAT0000583::MIMAT0000584::M			+	+	+	
4373041	000550	UCCGUCUCAGUUACUUUAUAGCC	hsa-miR-340	hsa-miR-340::mmu-i	MIMAT0000585::MIMAT0000586::M			+	+	+	
4373040	000551	UCUCACACAGAAAUCGCACCCGUC	hsa-miR-342	hsa-miR-342::mmu-i	MIMAT0000589::MIMAT0000590::M			+	+	+	
4373035	000554	UUAUCAGAAUCUCCAGGGGUAC	hsa-miR-361	bta-miR-361::hsa-mi	MIMAT0000703::MIMAT0000704::M			+	+	+	
4373028	000563	UUAUAAUACAACCUGAUAAGUG	hsa-miR-374	hsa-miR-374	MIMAT0000727			+			
4373022	000569	UAUGUAAUAUGGUCCACAUCUU	hsa-miR-380-3p	hsa-miR-380-3p	MIMAT0000735			+			
4373016	000575	CUGGACUUGGAGUCAGAAGGCC	hsa-miR-422b	hsa-miR-422b::mmu	MIMAT0000732::MIMAT0003151			+	+		
4373009	000577	UGAGGUAGUAAGUUGUAUUGUU	hsa-miR-98	age-miR-98::bta-miF	MIMAT0000096::MIMAT0000545::M			+	+	+	
4373179	000596	ACUAGACUGAAGCUCCUUGAGG	hsa-miR-151	hsa-miR-151	MIMAT0000757			+			
4373271	000597	UUUGGCAAUGGUAGAACUCACA	hsa-miR-182	dre-miR-182::hsa-m	MIMAT0000211::MIMAT0000259::M			+	+		
4373200	000603	CUGGACUUAGGGUCAGAAGGCC	hsa-miR-422a	hsa-miR-422a	MIMAT0001339			+			
4373185	001010	AACUGGCCCUCAAAGUCCCGCUUU	hsa-miR-193b	hsa-miR-193b	MIMAT0002819			+			
4373194	001020	UAAUGCCCCUAAAAAUCCUUAU	hsa-miR-365	cfa-miR-365::dre-mil	MIMAT0000710::MIMAT0000711::M			+	+	+	
4373203	001024	UAAUACUGUCUGGUAAAACCGU	hsa-miR-429	hsa-miR-429	MIMAT0001536			+			
4373216	001038	AGUGGGGAACCCUUCCAUGAGGA	hsa-miR-491	hsa-miR-491	MIMAT0002807			+			
4373221	001042	AUUACAUGGCCAAUCUC	hsa-miR-496	hsa-miR-496	MIMAT0002818			+			
4373225	001046	AUGCACCUGGGCAAGGAUUCUG	hsa-miR-500	hsa-miR-500	MIMAT0002871			+			
4373202	001104	AUCGGGAAUGUCGUGUCCGCC	hsa-miR-425	hsa-miR-425-3p::mr	MIMAT0001	hsa-miR-425(8.0)		+	+		
4373227	001109	AUCCUUGCUAUCUGGGUGCUA	hsa-miR-502	hsa-miR-502	MIMAT0002873			+			
4373381	001093	CGCAAGGAUGACACGCAAAUUCGUG.	RNU6B	RNU6B	NR 002752	U6	+	+			
4373164	000382	UGAGGUAGUAGAUUGUAUAGUU	hsa-let-7f	bta-let-7f::dre-let-7f::	MIMAT000(	mmu-let-7f(5.0)		+		+	
4373121	000391	UAGCAGCACGUAAAUAUUGGCG	hsa-miR-16	age-miR-16::ggo-mil	MIMAT0000069::MIMAT0000527::M			+	+	+	
4373079	000398	AAGCUGCCAGUUGAAGAACUGU	hsa-miR-22	age-miR-22::hsa-mil	MIMAT0000077::MIMAT0000531::M			+	+	+	
4373074	000399	AUCACAUUGCCAGGGAUUUCC	hsa-miR-23a	age-miR-23a::ggo-m	MIMAT0000078::MIMAT0000532::M			+	+	+	
4373065	000412	UAGCACCAUCUGAAAUCGGUU	hsa-miR-29a	hsa-miR-29a::mmu-i	MIMAT0000086::MIMAT0000535::M			+	+	+	
4373289	000415	UAGCACCAUUUGAAAUCGGU	hsa-miR-29c	gga-miR-29c::hsa-m	MIMAT0000536::MIMAT0000681::M			+	+	+	
4373048	000424	GUGCAUUGUAGUUGCAUUG	hsa-miR-33	gga-miR-33::ggo-mil	MIMAT0000091::MIMAT0000667::M			+	+	+	
4373008	000435	AACCCGUAGAUCCGAUCUUGUG	hsa-miR-99a	dre-miR-99::gga-miF	MIMAT0000097::MIMAT0000820::M			+		+	
4373007	000436	CACCCGUAGAACCGACCUUGCG	hsa-miR-99b	hsa-miR-99b::mmu-i	MIMAT0000132::MIMAT0000689::M			+	+	+	
4373157	000441	UCAAAUGCUCAGACUCCUGU	hsa-miR-105	ggo-miR-105::hsa-m	MIMAT0000102::MIMAT0002116::M			+			
4373155	000442	UAAAGUGCUGACAGUGCAGAU	hsa-miR-106b	age-miR-106b::ggo-	MIMAT0000386::MIMAT0000680::M			+	+	+	
4373151	000445	UGGAGUGUGACAAUGGUGUUUGU	hsa-miR-122a	gga-miR-122a::hsa-i	MIMAT000(	gga-miR-122a-1(5.0)::gç		+	+	+	
4373150	000446	UUAAGGCACGCGGUGAAUGCCA	hsa-miR-124a	age-miR-124a::gga-	MIMAT000(	mmu-miR-124a(5.0)		+		+	
4378064	000450	UCGUACCGUGAGUAAUAAUGC	hsa-miR-126	dre-miR-126::fru-miF	MIMAT000(	gga-miR-126(8.1)::mmu		+	+	+	

4373145	000454	CAGUGCAAUGUUAAAAGGGCAU	hsa-miR-130a	dre-miR-130a::hsa-r	MIMAT0000141::MIMAT0000425::M		+	+	+
4373144	000456	CAGUGCAAUGAUGAAAGGGCAU	hsa-miR-130b	hsa-miR-130b::mmu	MIMAT0000387::MIMAT0000691::M		+	+	+
4373138	000462	AGUGGUUUUACCCUAUGGUAG	hsa-miR-140	gga-miR-140::hsa-m	MIMAT000(	mmu-miR-140(5.0)	+		+
4373134	000466	UGAGAUGAAGCACUGUAGCUCA	hsa-miR-143	ggo-miR-143::hsa-m	MIMAT0000247::MIMAT0000435::M		+	+	+
4373133	000467	GUCCAGUUUUCCCAGGAAUCCCUU	hsa-miR-145	ggo-miR-145::hsa-m	MIMAT0000157::MIMAT0000437::M		+	+	+
4378066	000483	UGGUUCUAGACUUGCCAACUA	hsa-miR-182#	dre-miR-182*::fru-mi	MIMAT0000260::MIMAT0001272::M		+		
4378067	000488	GUGCCUACUGAGCUGAUAUCAGU	hsa-miR-189	hsa-miR-189::mml-n	MIMAT0000079::MIMAT0000218::M		+	+	+
4373105	000494	UAGCAGCACAGAAAUAUUGGC	hsa-miR-195	ggo-miR-195::hsa-m	MIMAT0000225::MIMAT0000461::M		+	+	+
4378069	000502	UAACACUGUCUGGUAACGAUGU	hsa-miR-200a	dre-miR-200a::fru-m	MIMAT0000519::MIMAT0000682::M		+	+	+
4378070	000529	UAAGUGCUUCCAUGUUUUGGUGA	hsa-miR-302a	hsa-miR-302a::mmu	MIMAT000(	hsa-miR-302(3.1)	+	+	
4378071	000531	UAAGUGCUUCCAUGUUUUAGUAG	hsa-miR-302b	gga-miR-302b::hsa-i	MIMAT000(	mmu-miR-302b(8.1)	+		
4373276	000532	ACUUUAACAUGGAAGUGCUUUCU	hsa-miR-302b#	hsa-miR-302b*	MIMAT0000714		+		
4378072	000533	UAAGUGCUUCCAUGUUUCAGUGG	hsa-miR-302c	gga-miR-302c::hsa-i	MIMAT0000717::MIMAT0003359		+		
4373032	000557	AAUAAUACAUGGUUGAUCUUU	hsa-miR-369-3p	bta-miR-369-3p::hsa	MIMAT000(	hsa-miR-369(6.0)	+		
4373026	000565	AUCAUAGAGGAAAAUCCACGU	hsa-miR-376a	hsa-miR-376a	MIMAT0000729		+		
4373021	000570	UGGUUGACCAUAGAACAUGCGC	hsa-miR-380-5p	bta-miR-380-5p::hsa	MIMAT0000734::MIMAT0000744::M		+	+	
4378074	000584	UAAAGCUAGAUAACCGAAAGU	hsa-miR-9#	dre-miR-9*::hsa-miR	MIMAT0000143::MIMAT0000442::M		+	+	
4373170	000589	UCACAGUGAACCGGUCUCUUUC	hsa-miR-128b	hsa-miR-128b::mmu	MIMAT0000675::MIMAT0000676::M		+	+	+
4373171	000590	CUUUUUGCGGUCUGGGCUUGC	hsa-miR-129	hsa-miR-129::xtr-mil	MIMAT0000242::MIMAT0003590		+		
4373181	000598	UGGAGAGAAAGGCAGUUC	hsa-miR-185	hsa-miR-185::mmu-i	MIMAT0000214::MIMAT0000455::M		+	+	+
4373184	001009	UAAGGUGCAUCUAGUGCAGUUA	hsa-miR-18b	bta-miR-18b::gga-m	MIMAT0001141::MIMAT0001412::M		+		
4378075	001012	AGAGGUAUAGGGCAUGGGAAAA	hsa-miR-202	hsa-miR-202	MIMAT0002811		+		
4373274	001013	UUUCCUAUGCAUAUACUUCUUU	hsa-miR-202#	gga-miR-202*::hsa-r	MIMAT0002810::MIMAT0003354		+		
4373189	001015	UAUGUGGGAUGGUAAACCGCUU	hsa-miR-299-3p	hsa-miR-299-3p	MIMAT0000687		+		
4373199	001023	ACUUCACCUGGUCCACUAGCCGU	hsa-miR-412	hsa-miR-412::mmu-i	MIMAT0001094::MIMAT0002170::M		+	+	+
4378076	001027	CUGGAUGGCUCCUCCAUGUCU	hsa-miR-432#	hsa-miR-432*	MIMAT0002815		+		
4373176	001096	UCUACAGUGCACGUGUCU	hsa-miR-139	hsa-miR-139::lla-miF	MIMAT0000250::MIMAT0000656::M		+	+	+
4373191	001101	AACACACCUGGUUAACCUCUUU	hsa-miR-329	hsa-miR-329	MIMAT0001629		+		
4373196	001102	AUCAUAGAGGAAAAUCCAUGUU	hsa-miR-376b	hsa-miR-376b	MIMAT0002172		+		
4373238	001145	CACUCAGCCUUGAGGGCACUUUC	hsa-miR-512-5p	hsa-miR-512-5p	MIMAT0002822		+		
4378092	001273	AAUCCUUGGAACCUAGGUGUGAGU	hsa-miR-362	hsa-miR-362	MIMAT0000705		+		
4378093	001274	AAUAUAACACAGAUGGCCUGU	hsa-miR-410	hsa-miR-410	MIMAT0002171		+		
4373123	000389	UAGCAGCACAUAAUGGUUUGUG	hsa-miR-15a	age-miR-15a::ggo-m	MIMAT000(	gga-miR-15a(8.1)	+	+	
4373122	000390	UAGCAGCACAUCAUGGUUUACA	hsa-miR-15b	age-miR-15b::bta-m	MIMAT0000124::MIMAT0000417::M		+	+	+
4373071	000403	CAUUGCACUUGUCUCGGUCUGA	hsa-miR-25	bta-miR-25::dre-miR	MIMAT0000081::MIMAT0000652::M		+	+	+
4373058	000421	UGUAAACAUCCUUGACUGGA	hsa-miR-30e-5p	hsa-miR-30e-5p::mr	MIMAT000(	gga-miR-30e(8.1)::hsa-r	+	+	+
4373010	000434	UUUGGCACUAGCACAUUUUUGC	hsa-miR-96	ggo-miR-96::hsa-mil	MIMAT0000095::MIMAT0002784::M		+		
4373272	000498	CCCAGUGUUCAGACUACCUGUUC	hsa-miR-199a	dre-miR-199::fru-miF	MIMAT000(	dre-miR-199a(6.0)::gga-	+	+	+
4378068	000499	UACAGUAGUCUGCACAUUGGUU	hsa-miR-199a#	dre-miR-199*::hsa-n	MIMAT0000230::MIMAT0000232::M		+	+	
4373100	000500	CCCAGUGUUUAGACUAUCUGUUC	hsa-miR-199b	bta-miR-199b::hsa-n	MIMAT0000263::MIMAT0003821::M		+		
4373080	000522	UGAUUGUCCAAACGCAAUUCU	hsa-miR-219	gga-miR-219::ggo-m	MIMAT000(	dre-miR-219(6.0)	+	+	+
4373054	000538	GCACAUUACACGGUCGACCUCU	hsa-miR-323	hsa-miR-323::mmu-i	MIMAT0000550::MIMAT0000551::M		+	+	+
4373043	000548	UCCAGCAUCAGUGAUUUUGUUGA	hsa-miR-338	hsa-miR-338::mdo-n	MIMAT0000581::MIMAT0000582::M		+	+	+
4373033	000556	ACAUAGAGGAAAUUCCACGUUU	hsa-miR-368	hsa-miR-368	MIMAT0000720		+		
4378073	000561	GAAGUGCUUCGAUUUUGGGGUGU	hsa-miR-373	hsa-miR-373	MIMAT0000726		+		
4373279	000562	ACUCAAAAUGGGGGCGCUUUCC	hsa-miR-373#	hsa-miR-373*	MIMAT0000725		+		
4373019	000572	GAAGUUGUUCGUGGUGGAUUCG	hsa-miR-382	hsa-miR-382::mmu-i	MIMAT0000737::MIMAT0000747		+	+	
4373201	000604	CAGCAGCAAUUCAUGUUUUGAA	hsa-miR-424	hsa-miR-424	MIMAT0001341		+		
4373206	001029	UUGCAUAUGUAGGAUGUCCCAU	hsa-miR-448	cfa-miR-448::hsa-mi	MIMAT0001532::MIMAT0001533::M		+	+	+
4373208	001031	UUUUUGCGAUGUGUUCCUAAUA	hsa-miR-450	cfa-miR-450::hsa-mi	MIMAT0001545::MIMAT0001546::M		+	+	
4373281	001032	UGUUUGCAGAGGAAACUGAGAC	hsa-miR-452	hsa-miR-452::mmu-i	MIMAT0001635::MIMAT0001637		+	+	
4378077	001033	UCAGUCUCAUCUGCAAAGAAG	hsa-miR-452#	hsa-miR-452*	MIMAT0001636		+		
4373210	001034	GAGGUUGUCCGUGGUGAGUUCG	hsa-miR-453	hsa-miR-453	MIMAT0001630		+		
4373215	001037	CAACCUGGAGGACUCCAUGCUG	hsa-miR-490	gga-miR-490::hsa-m	MIMAT0002806::MIMAT0003366::M		+	+	
4373217	001039	AGGACCUGCGGGACAAGAUUCUU	hsa-miR-492	hsa-miR-492	MIMAT0002812		+		
4373218	001040	UUGUACAUGGUAGGCUUUCAUU	hsa-miR-493	hsa-miR-493-5p	MIMAT0002	hsa-miR-493(7.1)	+		
4373228	001048	UAGCAGCGGGAACAGUUCUGCAG	hsa-miR-503	hsa-miR-503	MIMAT0002874		+		
4373230	001049	GUCAACACUUGCUGGUUUCCUC	hsa-miR-505	hsa-miR-505	MIMAT0002876		+		
4373232	001051	UUUUGCACCUUUUGGAGUGAA	hsa-miR-507	hsa-miR-507	MIMAT0002879		+		
4373209	001105	AAACCGUUACCAUUACUGAGUUU	hsa-miR-451	gga-miR-451::hsa-m	MIMAT0001631::MIMAT0003775		+		
4373213	001106	CCCAGAUAAUGGCACUCUCAA	hsa-miR-488	hsa-miR-488	MIMAT0002804		+		
4373229	001110	AGACCCUGGUCUGCACUCUAU	hsa-miR-504	hsa-miR-504	MIMAT0002875		+		
4378078	001113	CCUCUAGAUGGAAGCACUGUCU	hsa-miR-517#	hsa-miR-517*	MIMAT0002851		+		
4373250	001115	AAAGUGCAUCCUUUUAGAGGUUU	hsa-miR-519b	hsa-miR-519b	MIMAT0002837		+		
4378080	001126	AAAGUGCUUCCUUUUAGAGGC	hsa-miR-526b#	hsa-miR-526b*	MIMAT0002836		+		
4373239	001146	UUCACAGGGAGGUGUCAUUUAU	hsa-miR-513	hsa-miR-513	MIMAT0002877		+		
4378082	001158	UCUCUGGAGGGAAGCACUUUCUG	hsa-miR-518c#	hsa-miR-518c*	MIMAT0002847		+		
4378083	001161	AAAGCGCUUCUCUUUAGAGGA	hsa-miR-518f	hsa-miR-518f	MIMAT0002842		+		
4373251	001163	AAAGUGCAUCUUUUUAGAGGAU	hsa-miR-519c	hsa-miR-519c	MIMAT0002832		+		
4373266	001164	CAAAGUGCCUCCCUUUAGAGUGU	hsa-miR-519d	hsa-miR-519d	MIMAT0002853		+		
4373267	001165	AAAGUGCCUCCUUUUAGAGUGU	hsa-miR-519e	hsa-miR-519e	MIMAT0002829		+		
4373245	001171	AAAAUGGUUCCCUUUAGAGUGUU	hsa-miR-522	hsa-miR-522	MIMAT0002868		+		
4373260	001172	AACGCGCUUCCCUAUAGAGGG	hsa-miR-523	hsa-miR-523	MIMAT0002840		+		
4378087	001173	GAAGGCGCUUCCCUUUGGAGU	hsa-miR-524	hsa-miR-524	MIMAT0002850		+		
4378095	001277	GUCAUACACGGCUCUCCUCUCU	hsa-miR-485-3p	hsa-miR-485-3p	MIMAT0002176		+		
4378096	001278	UCCUGUACUGAGCUGCCCCGAG	hsa-miR-486	hsa-miR-486::mmu-i	MIMAT0002177::MIMAT0003130		+	+	
4378099	001281	CAUCUGGAGGUAAGAAGCACUUU	hsa-miR-516-5p	hsa-miR-516-5p	MIMAT0002859		+		
4378104	001287	GGUAGAUUCUCCUUCUAUGAG	hsa-miR-376a#	hsa-miR-376a*::mm	MIMAT0003197::MIMAT0003386::M		+	+	+
4378105	001288	UCGGGGAUCAUCAUGUCACGAG	hsa-miR-542-5p	hsa-miR-542-5p	MIMAT0003340		+		
4380918	001289	AUCAGCAAACAUUUAUUGUGUG	hsa-miR-545	hsa-miR-545	MIMAT0003165		+		
4380919	001290	AUUCUGCAUUUUUAGCAAGU	hsa-miR-544	hsa-miR-544	MIMAT0003164		+		
4380920	001510	AAUAUUAUACAGUCAACCUCU	hsa-miR-656	hsa-miR-656	MIMAT0003332		+		
4380921	001511	UGACAACUAUGGAUGAGCUCU	hsa-miR-549	hsa-miR-549	MIMAT0003333		+		
4380922	001512	GGCAGGUUCUCACCCUCUCUAGG	hsa-miR-657	hsa-miR-657	MIMAT0003335		+		
4380923	001513	GGCGGAGGGAAGUAGGUCCGUUGGU	hsa-miR-658	hsa-miR-658	MIMAT0003336		+		
4380925	001515	UACCCAUUGCAUAUCGGAGUUG	hsa-miR-660	hsa-miR-660	MIMAT0003338		+		
4380926	001516	AAUGACACGAUCACUCCCGUUGA	hsa-miR-425-5p	hsa-miR-425-5p::xtr-	MIMAT0003393::MIMAT0003640		+		
4380927	001517	AAUGGCGCCACUAGGGUUGUGCA	hsa-miR-652	hsa-miR-652::mmu-i	MIMAT0003322::MIMAT0003711		+	+	
4380928	001518	CAUGCCUUGAGUGUAGGACCGU	hsa-miR-532	bta-miR-532::hsa-mi	MIMAT0002888::MIMAT0002889::M		+	+	
4380929	001519	GCGACCCACUCUUGGUUUCCA	hsa-miR-551a	hsa-miR-551a	MIMAT0003214		+		
4380930	001520	AACAGGUGACUGGUUAGACAA	hsa-miR-552	hsa-miR-552	MIMAT0003215		+		
4380931	001521	AAAACGGUGAGAUUUUGUUUU	hsa-miR-553	hsa-miR-553	MIMAT0003216		+		
4380932	001522	GCUAGUCCUGACUCAGCCAGU	hsa-miR-554	hsa-miR-554	MIMAT0003217		+		
4380933	001523	AGGGUAAGCUGAACCUCUGAU	hsa-miR-555	hsa-miR-555	MIMAT0003219		+		
4380939	001529	AAAGUAGCUGUACCAUUUGC	hsa-miR-562	hsa-miR-562	MIMAT0003226		+		
4380940	001530	AGGUUGACAUACGUUUCCC	hsa-miR-563	hsa-miR-563	MIMAT0003227		+		
4380941	001531	AGGCACGGUGUCAGCAGGC	hsa-miR-564	hsa-miR-564	MIMAT0003228		+		
4380942	001532	GGCUGGCUCGCGAUGUCUGUUU	hsa-miR-565	hsa-miR-565	MIMAT0003229		+		
4380943	001533	GGGCGCCUGUGAUCCCAAC	hsa-miR-566	hsa-miR-566	MIMAT0003230		+		
4380945	001535	GCGACCCAUACUUGGUUUCAG	hsa-miR-551b	hsa-miR-551b	MIMAT0003233		+		
4380946	001536	AGUUAAUGAAUCCUGGAAAGU	hsa-miR-569	hsa-miR-569	MIMAT0003234		+		
4380947	001537	GAAAACAGCAAUUACCUUUGCA	hsa-miR-570	hsa-miR-570	MIMAT0003235		+		
4380948	001538	CAAAACUGGCAAUUACUUUUGC	hsa-miR-548a	hsa-miR-548a	MIMAT0003251		+		

4380949	001539	UAUGCAUUGUAUUUUUAGGUCC	hsa-miR-586	hsa-miR-586	MIMAT0003252			+		
4380950	001540	UUUCCAUAGGUGAUGAGUCAC	hsa-miR-587	hsa-miR-587	MIMAT0003253			+		
4380951	001541	CAAGAACCUCAGUUGCUUUUGU	hsa-miR-548b	hsa-miR-548b	MIMAT0003254			+		
4380952	001542	UUGGCCACAAUGGGUUAGAAC	hsa-miR-588	hsa-miR-588	MIMAT0003255			+		
4380953	001543	UCAGAACAAAUGCCGGUUCCCAGA	hsa-miR-589	hsa-miR-589	MIMAT0003256			+		
4380954	001544	UGUCUUACUCCCUCAGGCACAU	hsa-miR-550	hsa-miR-550	MIMAT0003257			+		
4380955	001545	AGACCAUGGGUUCUCAUUGU	hsa-miR-591	hsa-miR-591	MIMAT0003259			+		
4380957	001547	AGGCACCAGCCAGGCAUUGCUCAGC	hsa-miR-593	hsa-miR-593	MIMAT0003261			+		
4380958	001548	CCCAUCUGGGGUGGCCUGUGACUUU	hsa-miR-594	hsa-miR-594	MIMAT0003262			+		
4380959	001550	AAGCCUGCCCGGCUCCUCGGG	hsa-miR-596	hsa-miR-596	MIMAT0003264			+		
4380960	001551	UGUGUCACUCGAUGACCACUGU	hsa-miR-597	hsa-miR-597	MIMAT0003265			+		
4380961	001553	ACAGUCUGCUGAGGUUGGAGC	hsa-miR-622	hsa-miR-622	MIMAT0003291			+		
4380962	001554	GUUGUGUCAGUUUAUCAAAC	hsa-miR-599	hsa-miR-599	MIMAT0003267			+		
4380963	001556	ACUUACAGACAAGAGCCUUGCUC	hsa-miR-600	hsa-miR-600	MIMAT0003268			+		
4380964	001557	UAGUACCAGUACCUUGUGUUCA	hsa-miR-624	hsa-miR-624	MIMAT0003293			+		
4380965	001558	UGGUCUAGGAUUGUUGGAGGAG	hsa-miR-601	hsa-miR-601	MIMAT0003269			+		
4380966	001559	AGCUGUCUGAAAAUGUCUU	hsa-miR-626	hsa-miR-626	MIMAT0003295			+		
4380969	001562	GUUCUCCCAACGUAAGCCCAGC	hsa-miR-629	hsa-miR-629	MIMAT0003298			+		
4381007	001604	UUUAGGAUAAGCUUGACUUUUG	hsa-miR-651	hsa-miR-651	MIMAT0003321			+		
4381008	001605	CAAAAACCACAGUUUCUUUUGC	hsa-miR-548d	hsa-miR-548d	MIMAT0003323			+		
4380917	001283	CGGGUGGAUCACGAUGCAAUUU	hsa-miR-363#	hsa-miR-363*::rno-n	MIMAT0003209::MIMAT0003385::M			+		+
4378102	001285	AAUCGUACAGGGUCAUCCACUU	hsa-miR-487b	bta-miR-487b::hsa-r	MIMAT0003180::MIMAT0003847			+		
4380924	001514	CUUGGUUCAGGGAGGGUCCCCA	hsa-miR-659	hsa-miR-659	MIMAT0003337			+		
4380934	001524	GAUGAGCUCAUUGUAAUAUG	hsa-miR-556	hsa-miR-556	MIMAT0003220			+		
4380936	001526	UGAGCUGCUGUACCAAAAU	hsa-miR-558	hsa-miR-558	MIMAT0003222			+		
4380967	001560	GUGAGUCUCUAAGAAAAGAGGA	hsa-miR-627	hsa-miR-627	MIMAT0003296			+		
4380970	001563	AGUAUUCUGUACCAGGGAAGGU	hsa-miR-630	hsa-miR-630	MIMAT0003299			+		
4380971	001564	AGACCUGGCCCAGACCUCAGC	hsa-miR-631	hsa-miR-631	MIMAT0003300			+		
4380972	001566	CACACACUGCAAUUACUUUUGC	hsa-miR-603	hsa-miR-603	MIMAT0003271			+		
4380974	001569	AAACUACUGAAAAUCAAAGAU	hsa-miR-606	hsa-miR-606	MIMAT0003274			+		
4380975	001570	GUUCAAAUCCAGAUCUAUAAC	hsa-miR-607	hsa-miR-607	MIMAT0003275			+		
4380976	001571	AGGGGUGGUGUUGGGACAGCUCCGU	hsa-miR-608	hsa-miR-608	MIMAT0003276			+		
4380978	001573	AGGGUGUUUCUCUCAUCUCU	hsa-miR-609	hsa-miR-609	MIMAT0003277			+		
4380979	001574	CUAAUAGUAUCUACCACAAUAAA	hsa-miR-633	hsa-miR-633	MIMAT0003303			+		
4380987	001583	AUCGCUGCGGUUGCGAGCGCUGU	hsa-miR-639	hsa-miR-639	MIMAT0003309			+		
4380989	001586	AGGAAUGUUCCUUCUUUGCC	hsa-miR-613	hsa-miR-613	MIMAT0003281			+		
4380990	001587	GAACGCCUGUUCUUGCCAGGUGG	hsa-miR-614	hsa-miR-614	MIMAT0003282			+		
4380991	001588	UCCGAGCCUGGGUCUCCCUCU	hsa-miR-615	hsa-miR-615	MIMAT0003283			+		
4380992	001589	ACUCAAAACCCUUCAGUGACUU	hsa-miR-616	hsa-miR-616	MIMAT0003284			+		
4380993	001590	CAAAAAUCUCAAUUACUUUUGC	hsa-miR-548c	hsa-miR-548c	MIMAT0003285			+		
4380994	001591	AGACUUCCCAUUUGAAGGUGGC	hsa-miR-617	hsa-miR-617	MIMAT0003286			+		
4380995	001592	GUCCCUCUCCAAAUGUGUCUUG	hsa-miR-642	hsa-miR-642	MIMAT0003312			+		
4380996	001593	AAACUCUACUUGUCCUUCUGAGU	hsa-miR-618	hsa-miR-618	MIMAT0003287			+		
4380999	001596	AGUGUGGCUUUCUUAGAGC	hsa-miR-644	hsa-miR-644	MIMAT0003314			+		
4381000	001597	UCUAGGCUGGUACUGCUGA	hsa-miR-645	hsa-miR-645	MIMAT0003315			+		
4381002	001599	AAGCAGCUGCCUCUGAGGC	hsa-miR-646	hsa-miR-646	MIMAT0003316			+		
4381003	001600	GUGGCUGCACUCACUUCCUUC	hsa-miR-647	hsa-miR-647	MIMAT0003317			+		
4381005	001602	AAACCUGUGUUGUUCAAGAGUC	hsa-miR-649	hsa-miR-649	MIMAT0003319			+		
4381006	001603	AGGAGGCAGCGCUCUCAGGAC	hsa-miR-650	hsa-miR-650	MIMAT0003320			+		
4381009	001606	UGCCUGGGUCUCUGGCCUGCGCGU	hsa-miR-661	hsa-miR-661	MIMAT0003324			+		
4381010	001607	UCCCACGUUGUGGCCCAGCAG	hsa-miR-662	hsa-miR-662	MIMAT0003325			+		
4381011	001608	AGGCAGUGUAUUGUUAGCUGGC	hsa-miR-449b	hsa-miR-449b	MIMAT0003327			+		
4381012	001609	UUGAAACAAUCUCUACUGAAC	hsa-miR-653	hsa-miR-653	MIMAT0003328			+		
4381013	001610	UAGUAGACCGUAUAGCGUACG	hsa-miR-411	hsa-miR-411	MIMAT0003329			+		
4381014	001611	UGGUGGGCCGCAGAACAUGUGC	hsa-miR-654	hsa-miR-654	MIMAT0003330			+		
4381017	001614	GUCCGCUCGGCGGUGGCCCA	hsa-miR-572	hsa-miR-572	MIMAT0003237			+		
4381020	001617	GAGCCAGUUGGACAGGAGC	hsa-miR-575	hsa-miR-575	MIMAT0003240			+		
4381021	001618	AUUCUAAUUUCUCCACGUCUUUG	hsa-miR-576	hsa-miR-576	MIMAT0003241			+		
4381022	001619	CUUCUUGUGCUCUAGGAUUGU	hsa-miR-578	hsa-miR-578	MIMAT0003243			+		
4381023	001620	AUUCAUUUGGUAUAAACCGCGAU	hsa-miR-579	hsa-miR-579	MIMAT0003244			+		
4381024	001621	UUGAGAAUGAUGAAUCAUUAGG	hsa-miR-580	hsa-miR-580	MIMAT0003245			+		
4381027	001625	UGGGCGUAUCUGUAUGCUA	hsa-miR-585	hsa-miR-585	MIMAT0003250			+		
4381028	001800	UAAUACUGCCUGGUAAUGAUGAC	hsa-miR-200b	hsa-miR-200b::mmu	MIMAT0000233::MIMAT0000318::M			+	+	+
4381032	001821	UCAGGCUCAGUCCCCUCCCGAU	hsa-miR-484	bta-miR-484::hsa-mi	MIMAT0002174::MIMAT0003127::M			+	+	
4381034	001823	AAGUGCUGUCAUAGCUGAGGUC	hsa-miR-512-3p	hsa-miR-512-3p	MIMAT0002823			+		
4373383	001006	GAUGACCCCAGGUAACUCUGAGUGU	RNU48	RNU48	NR_002745	U48	+	+		
4373384	001094	CCUGGAUGAUGAUAGCAAAUGCUGA	RNU44	RNU44	NR_002750	U44	+	+		
