# Supplementary material for: Identification of MicroRNAs as Potential Prognostic Markers in Ependymoma
Source: PLoS One. 2011 Oct 28;6(10):e25114. doi: 10.1371/journal.pone.0025114 (PMC3203863; doi:10.1371/journal.pone.0025114)
Supplement: Table S2 — Target identification for differentially expressed miRNAs in ependymomas based on GoMir, GeneGo, literature search and microarray gene expression analyses (p<0.05). (DOC) [file pone.0025114.s005.doc]

**Table S2:** Target identification for differentially expressed miRNAs in ependymomas based on GoMir, GeneGo, literature search and microarray gene expression analyses (p<0.05).

| **miRNA** | **Putative Target Gene(s)** | **Ependymomas**  **x**  **Normal controls*** | **P value** |
| --- | --- | --- | --- |
| miR-17-5p (up)** | RASSF2 (down) | 0.37 | 0.038*** |
| miR- 34a (up)** | CNTN2 (down)  VAMP2 (down) | 0.16  0.46 | 0.00018***  0.04*** |
| miR-383 (down)** | CTNNAL1 (up) | 2.67 | 0.0013*** |
| miR-485-5p (down)** | TGFbeta I (up)  TGFbeta II (up)  TGFbetai (up) | 3.3  4.0  13.0 | 0.0001***  0.04***  1.9E-09*** |

*Analyses based on normalization of ependymoma samples (n=21) against normal brain enriched for ependymal cells (n=6) using data from microarray gene expression.

****** Confirmed by individual assays.

*** Statistically significant according to t-test.
